# Rational Design and Experimental Analysis of Short-Oligonucleotide Substrate Specificity for Targeting Bacterial Nucleases

**DOI:** 10.1021/acs.jmedchem.1c00884

**Published:** 2021-08-30

**Authors:** Tania Jiménez, Juliana Botero, Dorleta Otaegui, Javier Calvo, Frank J. Hernandez, Eider San Sebastian

**Affiliations:** † Somaprobes S.L, Mikeletegi Pasealekua, 83, 20009 Donostia, Gipuzkoa, Spain; ‡ Applied Chemistry Department, University of the Basque Country (UPV/EHU), 20018 San Sebastián, Spain; § Center for Cooperative Research in Biomaterials (CIC biomaGUNE), Basque Research and Technology Alliance (BRTA), San Sebastian 20014, Spain; ∥ Wallenberg Center for Molecular Medicine (WCMM), 58185 Linköping, Sweden; ⊥ Department of Physics, Chemistry and Biology, Linköping University, 58185 Linköping, Sweden

## Abstract

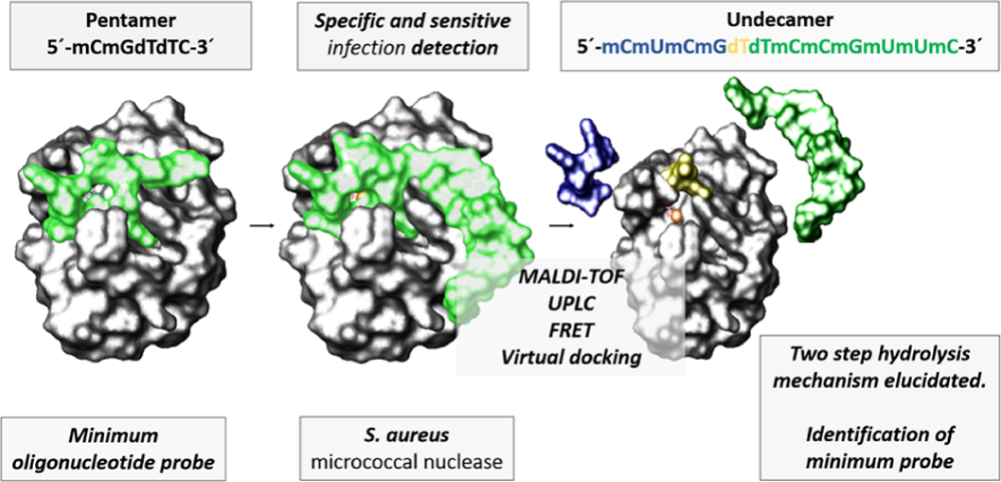

An undecamer oligonucleotide probe based on a pair of deoxythymidines flanked by several modified nucleotides is a specific and highly efficient biosensor for micrococcal nuclease (MNase), an endonuclease produced by *Staphylococcus aureus*. Herein, the interaction mode and cleavage process on such oligonucleotide probes are identified and described for the first time. Also, we designed truncated pentamer probes as the minimum-length substrates required for specific and efficient biosensing. By means of computational (virtual docking) and experimental (ultra-performance liquid chromatography–mass spectrometry and matrix-assisted laser desorption ionization time-of-flight) techniques, we perform a sequence/structure–activity relationship analysis, propose a catalytically active substrate–enzyme complex, and elucidate a novel two-step phosphodiester bond hydrolysis mechanism, identifying the cleavage sites and detecting and quantifying the resulting probe fragments. Our results unravel a picture of both the enzyme–biosensor complex and a two-step cleavage/biosensing mechanism, key to the rational oligonucleotide design process.

## 1 Introduction

Nucleic acids have been proven useful recognition molecules for the development of several diagnostic strategies, taking advantage of their flexibility to be adapted to various transduction mechanisms, such as fluorescence, electrochemistry, piezoelectric, and colorimetric mechanisms.^1^ In particular, the use of nuclease-activatable oligonucleotide probes (NAOPs) as biosensors has proven its potential as a groundbreaking solution in early diagnosis of serious infectious diseases.^2,3^ The detection of bacterial infection,^4−6^ malignant cells in biopsies of breast cancer,^7−9^ or food contamination by pathogenic bacteria^1^ has been previously reported to be feasible on the basis of their nuclease activity profile.^10−12^ A key aspect in the development of any diagnostic tool based on oligonucleotide probes is the necessity of designing probes which are both efficient and specific.^13^ Efficiency is directly related to the extent to which the target nuclease cleavages the synthetic substrate. In this respect, the oligonucleotide probe needs to be readily cleaved by the target nuclease even when the latter is in very low concentrations, such as in the early stages of infection. In addition, in order to avoid false positives in the diagnosis, a reliable biosensor needs to be resistant to cleavage by other nucleases that may be present in the sample under analysis.

**Figure 1:**
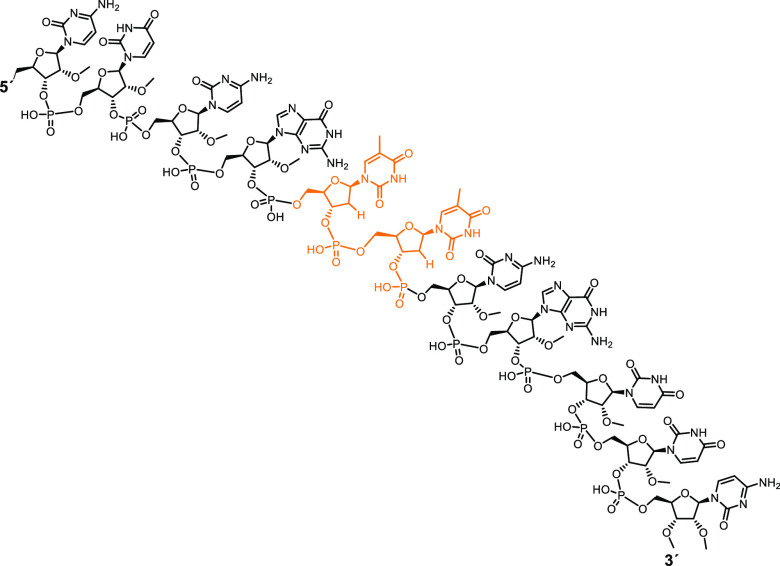
Chemical structure of the nude-dTdTprobe. FAM fluorophore and TQ2 quencher at the 5′- and 3′-ends, respectively, flank the sequence in the otherwise identical FRET-dTdT probe.

In this line, in a previous work,^4^ Hernandez et al. described the efficiency and specificity of a library of oligonucleotide probes in the detection of bacterial infection caused by *Staphylococcus aureus* (*S. aureus*) based on the activity of their secreted endonuclease, the micrococcal nuclease (MNase). In that study, the MNase activity was detected through a fluorescence resonance energy transfer (FRET) method, using specific oligonucleotide probes modified at the 5′-end with a fluorophore (fluorescein amidite, FAM) and at the 3′-end with a fluorophore quencher (tide quencher 2, TQ2), as depicted in Figure S1. Once the oligonucleotide is cleaved by the enzyme, the fluorophore is released, and the fluorescence intensity is quantified in order to estimate the nuclease activity. Among all the NAOPs tested,^4^ the one consisted of a pair of deoxythymidines (dTs) flanked by several 2′-*O*-methyl-modified nucleotides, 5′-mCmUmCmGdTdTmCmGmUmUmC-3′ [named the TTprobe in previous reports^4^ and hereafter the FRET-dTdTprobe when used in FRET experiments or the nude-dTdTprobe when used in ultra-performance liquid chromatography mass spectrometry (UPLC–MS) and matrix-assisted laser desorption ionization time-of-flight (MALDI-TOF) experiments; see Figure 1], noticeably exhibited the greatest sensitivity and specificity to be digested by MNase.^4^ The FRET-dTdTprobe has also been used to test *S. aureus* antibacterial susceptibility with high accuracy in comparison with the gold-standard broth microdilution method^14^ and very recently was also used as a 6-mer oligonucleotide linker to covalently functionalize a hydrogel coating with vancomycin, aiming to enable a rapid release of vancomycin within the periprosthetic implant infected with *S. aureus*.^15^

**Figure 2:**
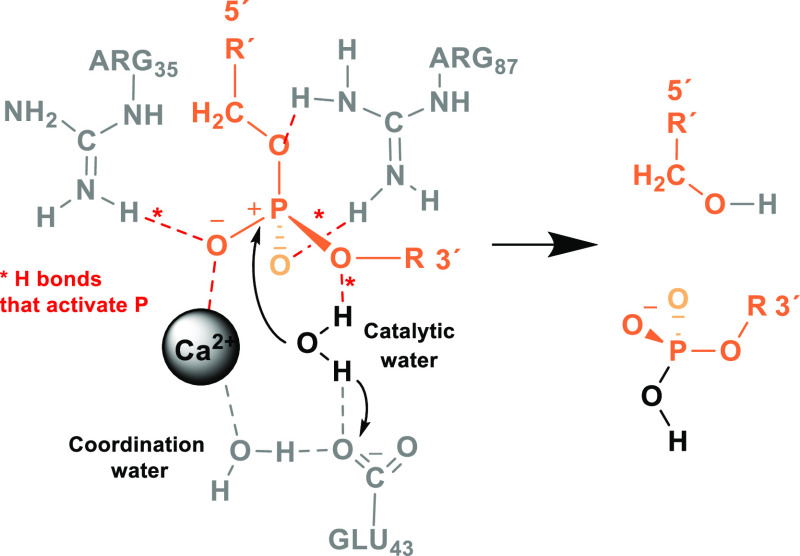
Schematic representation of the interactions that may be occurring in the catalytic complex formed between a phosphate group and key residues in the *S. aureus* MNase active site and cleavage fragments obtained upon hydrolysis.

The great advances in the nuclease detection technology reported so far lack a rational and atomic-level description of the interaction mode and catalytic reaction of the oligo–enzyme complex. An unprecedented description of the catalytic interactions between the said nude- or FRET-dTdTprobe and MNase is proposed here for the first time based on the coherent results obtained with the full-length oligomer and shorter pentameric sequences designed both for docking purposes and for the identification of the minimum-length required in the probe candidate. Computational and experimental techniques were used, which combined with the previous knowledge on the phosphodiester bond hydrolysis mechanism^16^ (Figure 2) have reported consistent results, providing a basic and essential understanding of the process. Importantly, we describe probe candidates with low molecular weights compared with that of previous reports^17−19^ that work efficiently as biosensors.

## 2 Experimental Section

### 2.1 Nuclease-Activatable Oligonucleotide Probes

All NAOPs were purchased at Biomers.net (Germany) with and without the FAM fluorophore at the 5′-end and TQ2 at the 3′-end. All of them were diluted in PBS +/+ (with CaCl_2_ and MgCl_2_) to a final concentration of 50 pmol/μL.

### 2.2 Bacterial Strain, Culture, and Supernatant Preparation for the Nuclease Activity Assay

A total of five bacterial strains were selected and purchased for the specificity test: *S. aureus* ATCC 29213, *Proteus mirabilis* ATCC 25933, *Klebsiella pneumoniae* ATCC 13883, *Pseudomonas aeruginosa* ATCC 10145, and *Streptococcus pneumoniae* ATCC 49619. Nutrient broth (NB), Trypticase soy broth (TSB), Todd Hewitt broth (THB) culture media, yeast extract (YE) supplement, and agar (A) were purchased from Fisher Scientific. To prepare pure culture supernatants, frozen bacterial strains ATCC 25933, ATCC 13883, and ATCC 10145 were precultured in nutrient agar (NB + A), while bacterial strain ATCC 29213 was precultured in Trypticase soy agar (TSB + A), and bacterial strain ATCC 49619 was precultured in Todd Hewitt agar supplemented with 2% YE (THB + A + YE) for 24 h at 37 °C. Thereafter, one individual colony of strains ATCC 25933, ATCC 13883, and ATCC 10145 was transferred to NB culture medium; ATCC 29213 was transferred to TSB culture medium; and ATCC 49619 was transferred to THB culture medium supplemented with 2% YE (THB + YE) and incubated at 37 °C overnight, shaking at 200 rpm. The cultures obtained were diluted 1:500 in fresh broth culture medium and cultured at 37 °C for 24 h, shaking at 200 rpm. Each culture was then centrifuged at 6000*g* for 20 min, and the supernatant was removed and used immediately or kept at 4 °C until experiments were performed.

### 2.3 Nuclease Activity Assays

The nuclease activity assays (NAAs) were performed using the standard conditions previously reported.^4^ NAA with MNase: MNase was purchased from Thermo Scientific (MNase 8000 U). 1 μL of NAOPs (50 pmol nuclease substrate) was mixed with 1 μL of MNase (1 U/μL) in 8 μL of PBS +/+ (supplemented with CaCl_2_ and MgCl_2_) and incubated at 37 °C for 60 min. Thereafter, the reaction was stopped by adding 295 μL of PBS-/- (GIBCO, without CaCl_2_ and MgCl_2_) supplemented with 10 mM EDTA (Thermo Fisher). Next, 95 μL of each sample was loaded in triplicate into a 96-well black plate (96F nontreated black microwell plate, Thermo Scientific). NAA with *bacterial supernatants*: 1 μL of NAOPs (50 pmol nuclease substrate) was mixed with 9 μL of the bacterial supernatant and incubated at 37 °C for 60 min. After the incubation, the reaction was stopped by adding 295 μL of PBS-/- supplemented with 10 mM EDTA. Then, 95 μL of each sample was loaded in triplicate into a 96-well black plate. Fluorescence intensity was measured with a fluorescence microplate reader (Synergy HT, BioTek and Synergy neo2, BioTek) using the filter settings for FAM (excitation/emission 494/521 nm). Three independent experiments were performed for each sample. The results are plotted as the mean ± SD (*n* = 3) of the fluorescence intensity given in arbitrary units (a.u.), as well as the activation ratio fold (ARF), calculated according to eq 1
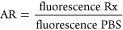
1where fluorescence Rx is the amount of arbitrary units of fluorescence obtained for the enzymatic reaction of MNase with the probe and fluorescence PBS is the amount of arbitrary units of fluorescence obtained for the control solution of the same probe only in PBS +/+ as the baseline autofluorescence of the probe.

### 2.4 NAAs for MALDI Experiments

After some optimization procedures to set the most favorable conditions to perform the MALDI-MS experiments, the standard conditions defined for the NAA were as follows: a mixture of 1 μL of MNase (1 U/uL) reacting with 1 μL of the dTdTprobe NAOP (with or without FAM and TQ2) at a concentration of 50 pmol in 8 μL of PBS +/+ and incubated at 37 °C for 60 min. Thereafter, the reaction was stopped thermically by freezing it in dry ice and was kept frozen in an ultrafreezer at −80 °C until the MALDI assays were performed. The sample preparation was carried out by the deposition of 0.5 μL of the sample (the NAA reaction) directly onto a polished stainless-steel plate (Bruker Daltonics, Bremen, Germany) and mixed with 0.5 μL of the matrix solution, composed of a dissolution of 10 mg of 2,4,6-trihydroxyacetophenone and 5 mg of diammonium citrate in 1 mL of deionized water. MALDI-TOF-MS analysis was performed using an UltrafleXtreme III time-of-flight mass spectrometer equipped with a Nd:YAG laser (Smartbeam II, 355 nm, 1 kHz) and controlled by Flex Control 3.3 software (Bruker Daltonics, Bremen, Germany). The acquisitions were carried out in the positive-ion linear mode at a laser frequency of 1 kHz. The spectrum was acquired at 30% laser fluency and was recorded in the *m*/*z* range from 1500 to 5000. The deflector cutoff was set at *m*/*z* 1300, and the spectrum resulted from the accumulation of 3000 laser shots. All the spectra were analyzed using FlexAnalysis software 3.0 (Bruker Daltonics, Bremen, Germany).

### 2.5 LC–MS Analysis

After some optimization procedures to set the most favorable conditions to perform the UPLC–MS experiments, the standard conditions defined for the NAAs were as follows: 1 μL of MNase (20 U/μL) reacted with 1 μL of pent#4 (1 nmol nuclease substrate) in a 1.5 mL hplc vial, and 198 μL of PBS +/+ was added. The vial was then placed in the autosampler carousel set at 37 °C. Chromatography was performed in an Acquity UPLC system using an Acquity BEH C18 column (100 × 2.1 mm, 1.7 μm) from Waters (Milford, MA, USA). The samples were eluted using a flow rate of 300 μL min^–1^ and using as a mobile phase 100 mM triethylamine in water (A) and 100 mM triethylamine in acetonitrile/water (1:1) (B). The gradient method was as follows: 0–0.5 min at 95% A, 0.5–3.5 min to 50% A, 3.5–3.7 min to 1% A, 3.7–4.2 min at 1% A, 4.2–4.3 min to 95% A, and 4.3–5 min at 95% A. The UV detector wavelengths were set at 262 and 280 nm, and the injection volume was 10 μL. The MS detection was carried out using the electrospray ionization TOF mass spectrometer LCT Premier XE from Waters (Milford, MA, USA) with an electrospray ionization source, working in the negative/W mode. The MS range acquired was between *m*/*z* 100 and 2000. The capillary and cone voltages were set at 1000 and 100 V, respectively. For other parameters, the desolvation gas temperature was 350 °C, and the source temperature was 120 °C. The desolvation and cone gas rates were set at 600 and 30 L h^–1^, respectively. MassLynx v4.1 software was used to analyze the chromatograms and spectra (Waters, Milford, MA, USA).

### 2.6 Computational Studies

#### 2.6.1 Virtual Docking

Due to the limitations (maximum number of atoms) of the technique, virtual docking experiments were carried out with suitable molecular models of the nude dTdT probe. Virtual docking of several short-oligonucleotide probes to the MNase receptor structure was made with the Glide software package of Schrodinger Inc.^20^ A careful analysis (beyond the scope of this report) of the wide variety of structures deposited in the PDB for this nuclease pointed to PDB ID = 1SNC (deposited by Loll and Lattman^21^) as the most appropriate to perform the virtual docking study. The latter crystal structure consists of the ternary complex of residues 7–141 of staphylococcal nuclease, Ca^2+^, and the inhibitor thymidine-3′,5′-diphosphate (THP) refined at 1.65 Å.

#### 2.6.2 Receptor Preparation and Grid Generation

The structure of residues 7–141 of staphylococcal nuclease was downloaded from the protein data bank (PDB ID: 1SNC) and prepared with Schrödinger’s Protein Preparation Wizard suite in order to add the missing hydrogens, assign bond orders, create zero-order bonds to the Ca^2+^ metal, and optimize the H-bond network. The preprocessing was carried out with default methods, except that water molecules beyond 3 Å of any heavy atoms were removed. Minimization and refinement were performed to remove local clashes prior to further standard equilibration protocols. The resulting structure contained seven different water molecules, five of which establish key interactions with the cocrystallized THP ligand but are located far from the active site on the enzyme. In view of the latter, two alternative Glide grid files were generated for the MN receptor, differing in the amount of water molecules, using an enclosing box of ca. 46 Å centered on the Ca^2+^ atom, which properly covered the active site of the enzyme (see the Supporting Information for further details).

#### 2.6.3 Ligand Preparation and Docking

Pentameric oligonucleotides (ligands) to be screened were manually generated using the Build facility in Maestro 12.5 and prepared for docking using LigPrep 5^22^ with the OPLS_2005 force field. To set the ionization and tautomerization state of compounds at a pH range of 6–8, Epik v16207 was used, with a maximum number of 10 generated structures. The binding mode and affinity of the ligands were estimated using an extra precision Glide docking protocol, with and without positional NOE and metal coordination constraints.

### 2.7 Identifying Catalytically Relevant Poses

The hydrolysis of oligonucleotides promoted by MNase was proposed^16^ to proceed via the cleavage of a 5′-P-O bond to yield a free 5′-hydroxyl group and a terminal 3′-phosphate monoester group (see Figure 2). The hydrolysis of one single phosphate bond may consist of four consecutive steps involving a catalytic water molecule, three amino acids, and a Ca^2+^ ion. The efficiency and specificity of such hydrolysis depend on the nature of the substrate sequence for each nuclease.

According to the authors, hydrolysis may follow an SN2-type mechanism (see Figures 2 and S4), starting with the nucleophilic attack of the oxygen atom of a catalytic water molecule (HOH225) on the P atom of a Ca^2+^-coordinated phosphate group. The attack is believed to be favored upon the polarization of the coordinated phosphate group via an H-bond network governed by several coordination water molecules as well as residues ARG35 and ARG87. The latter residue was also proposed by the authors as being the acidic residue that transfers a proton to the 5′-oxygen atom of the phosphate group, yielding the final cleavage products composed of a 5′-OH fragment and a 3′-phosphate. Actually, the Ca^2+^ metal center cooperating with guanidinium residues showed a nearly 35 000-fold rate enhancement of DNA hydrolysis compared to that of the mutant without the guanidinium groups.^10^ Similar mechanisms have been reported for other endonucleases,^11^ even for the cleavage of double-stranded DNA. In the present work, catalytically relevant pentamer/MNase poses were identified among those which were characterized by (i) phosphate group coordination to the Ca^2+^ cation, accompanied by the establishment of key H-bonds with neighboring residues or water molecules that would polarize the phosphate group; (ii) coordination of a catalytic water molecule (HOH225) to a basic residue (such as GLU43) as well as to the 3′-*O* atom of the coordinated phosphate group; and (iii) H-bonding of an acidic residue (such as ARG 87) to the 5′-O atom of the Ca^2+^-coordinated phosphate group.

## 3 Results and Discussion

### 3.1 Digestion of NAOPs by MNase: Fluorescence Assay

**Figure 3:**
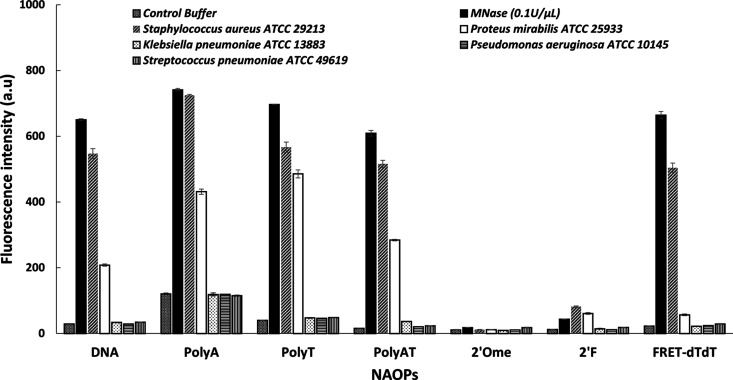
Screening of the nuclease activity profile of five pathogen strains and MNase (0.1 U/uL). Results reported as an increase in the mean fluorescence intensity as a result of the degradation of each NAOP. The bars represent the average of triplicate fluorescence measurements (±s.d).

Pathogen specificity of seven NAOPs (DNA, poly A, poly T, poly AT, 2′OMe, 2′F, and FRET-dTdT; see Table 1 for sequence details) was tested by an NAA carrying out the well-established FRET fluorescence method described before^4^ and using five different pathogen strains (*S. aureus*, *P. mirabilis*, *K. pneumoniae*, *P. aeruginosa,* and *S. pneumoniae*), as well as purified MNase (*C*_f_ 0.1 U/μL). NAOP incubation and digestion promoted fluorescence emission, which is reported below as the total fluorescence in arbitrary units (a.u) and the fluorescence intensity fold (or the ARF) with respect to the control (see Figure 3 and Table 1). As observed in this experiment, NAOPs made of DNA (unmodified), with codes DNA, poly A, poly T, and poly AT, were digested mainly by MNase, as well as by two distinct pathogen strains (*S. aureus* and *Proteus vulgaris*). For instance, DNA probe digestion provoked ARFs of 22.4 (±0.8), 18.9 (±0.8), and 7.2 (±0.3), respectively, with respect to the control buffer for the three mentioned bacterial strains. On the other hand, NAOPs poly A, poly T, and poly AT showed similar digestion profiles, with the poly AT probe showing the largest activation ratio among all. Due to the observed lack of specificity in the digestion of those NAOPs by *S. aureus*, they were discarded as molecular candidates for bacterial detection. On the contrary, two chemically modified NAOPs, 2′OMe and 2′ F, were largely resistant to be cleaved by all strains, emitting barely any fluorescence in any of the FRET experiments. In that respect, we conclude that the FRET-dTdTprobe was the only NAOP specifically digested by the *S. aureus* bacterial supernatant and pure MNase, with ARFs in comparison with the control buffer of 22.6(±1.3) and 29.7(±1.6), respectively. These results are in good agreement with our previous findings, confirming a highly efficient and specific degradation of the FRET-dTdTprobe (reported previously as the TTprobe).^4^ As required for the specific detection criterion, the FRET-dTdTprobe withstood digestion by all other bacterial strains, showing insignificant ARF fluorescence values of 2.5(±0.2), 1.1(±0.1), 1.1(±0.1), and 1.3(±0.1) when incubated with bacterial supernatants of *P. mirabilis*, *K. pneumoniae*, *P. aeruginosa,* and *S. pneumoniae* cultures, respectively.

**Table 1:** ARF Intensity of Fluorescence for the FRET-NAOPs, Obtained from Digestion Experiments Upon Incubation with Either Purified MNase or One of the Five Pathogen Strains Cultured

ARF intensity of fluorescence						
probe/strain	MNase	*S. aureus*	*P. mirabilis*	*K. pneumoniae*	*P. aeruginosa*	*S. pneumoniae*
DNA	5′-FAM-dTdCdTdCdGdTdAdCdGdTdTdC-3′-TQ2					
	22.4(±0.8)	18.9(±0.8)	7.2(±0.3)	1.2(±0.1)	1.0(±0.0)	1.2(±0.1)
poly A	5′-FAM-dAdAdAdAdAdAdAdAdAdAdAdA-3′-TQ2					
	6.2(±0.2)	6.0(±0.2)	3.6(±0.1)	1.0(±0.0)	1.0(±0.0)	1.0(±0.0)
poly T	5′-FAM-dTdTdTdTdTdTdTdTdTdTdTdT-3′-TQ2					
	17.6(±0.3)	14.3(±0.4)	12.2(±0.3)	12.2(±0.0)	12.2(±0.0)	12.2(±0.0)
poly AT	5′-FAM-dAdAdAdTdTdTdAdAdAdTdTdT-3′-TQ2					
	38.1(±0.5)	32.2(±0.7)	17.8(±0.2)	1.3(±0.0)	1.3(±0.0)	1.5(±0.0)
2′Ome	5′-FAM-mUmCmUmCmGmUmAmCmGmUmUmC-3′-TQ2					
	1.6(±0.16)	1.1(±0.1)	1.0(±0.1)	1.0(±0.1)	1.0(±0.1)	1.6(±0.2)
2′F	5′-FAM-fUfCfUfCfGfUfAfCfGfUfUfC-3′-TQ2					
	3.5(±0.2)	6.6(±0.3)	4.9(±0.3)	0.9(±0.1)	0.9(±0.1)	1.5(±0.1)
FRET-dTdT	5′-FAM-mCmUmCmGdTdTmCmGmUmUmC-3′-TQ2					
	29.7(±0.1.7)	22.6(±0.1.3)	2.5(±0.2)	1.1(±0.1)	1.1(±0.1)	1.3(±0.1)

### 3.2 Identification of the Cleavage Site(s) in the dTdTprobe by MS

The cleavage site(s) and origin of the great selectivity and efficiency of the hydrolysis of the described FRET-dTdTprobe by *S. aureus* MNase are summarized in the following lines, contributing therefore key knowledge for the improvement of the design of NAOPs that will specifically and efficiently target the detection of this or other specific nuclease activity. With that purpose, the degradation products (oligonucleotide fragments) generated upon the incubation of MNase with the nude-dTdTprobe were identified by MALDI-TOF spectrometry and virtual docking experiments.

**Figure 4:**
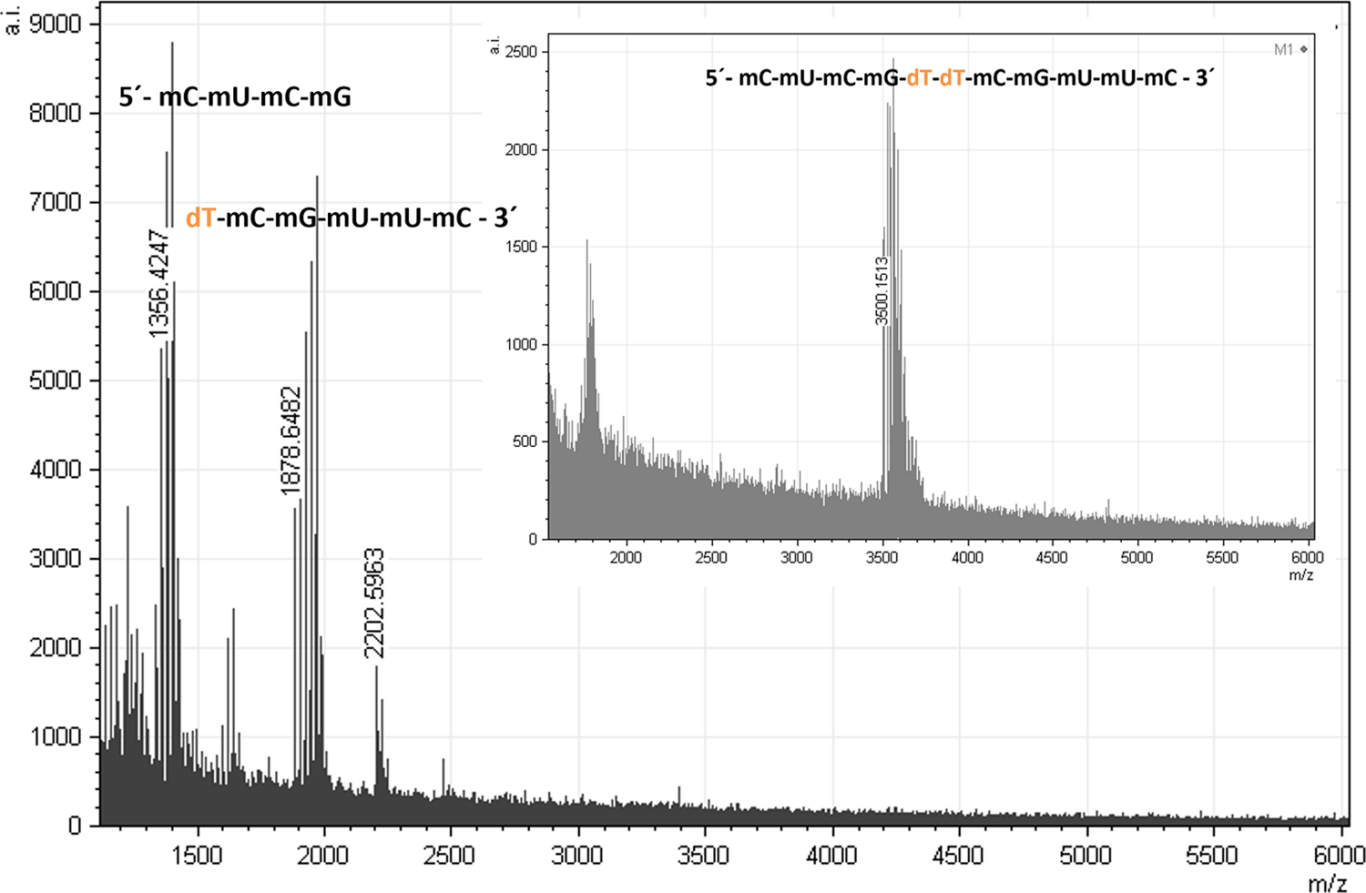
MALDI-TOF-MS spectrum of the digestion products of the nude-dTdTprobe after 1 h incubation with MNase. Starting construct (subpanel) and degradation fragments (panel).

Two alternative constructs of the said FRET-dTdTprobe were included in the study described below, that is, with (the FRET-dTdTprobe) and without (the nude-dTdTprobe) the FAM fluorophore at the 5′-end and the TQ2 quencher at the 3′-end, respectively. The FAM and TQ2 labels, necessary to detect and quantify the digestion of the probe in the FRET experiments, were first proven not to alter the cleavage site(s) or reaction efficiency. In this regard, upon incubation with MNase, digestion products of both the nude-dTdTprobe (MW: 3500.15 Da) and the FRET-dTdTprobe (MW: 4673.33 Da) were analyzed by means of MALDI-TOF experiments (Figures 4 and S2), which revealed that both probes undergo the same two scissions and generate equivalent oligonucleotide fragments. In the case of the nude-dTdTprobe, the starting construct of 3500.15 Da (subpanel in Figure 4) is cleaved into two detectable fragments (Figure 4), with masses *m*/*z*: 1356 and 1878 Da, compatible with fragments 5′-mCmUmCmG and dTmCmGmUmUmC-3′, respectively. Also, a signal consistent with dTdTmCmGmUmUmC-3′ was found as a minor product (2202 Da). As the first attempt, these fragments obtained imply that the cleavage occurs at the 5′ thymine position. Sequence 5′-mCmUmCmGdT was not observed under these reaction conditions. Thus, a third, low-mass (322.21 Da), and nondetectable fragment corresponding to single dT–OH is expected to be a product of the nuclease activity on the nude-dTdTprobe as well (note that the 5′-3′-nuclease activity of MNase promotes the cleavage of phosphodiester groups, generating the 3′-hydrolyzed phosphate ends (−PO_4_H_2_) and the protonated 5′-oxygen atoms in the leaving fragment). Analogous results were obtained in experiments ran with the FRET-dTdTprobe (see Figure S2), confirming the existence of two cleavage sites, and evidenced that the fluorophore and quencher used in the FRET experiments located at the 5′- and 3′-ends of the oligonucleotide sequences, respectively, do not alter the cleavage pattern and presumably the cleavage efficiency.

**Scheme 1:**
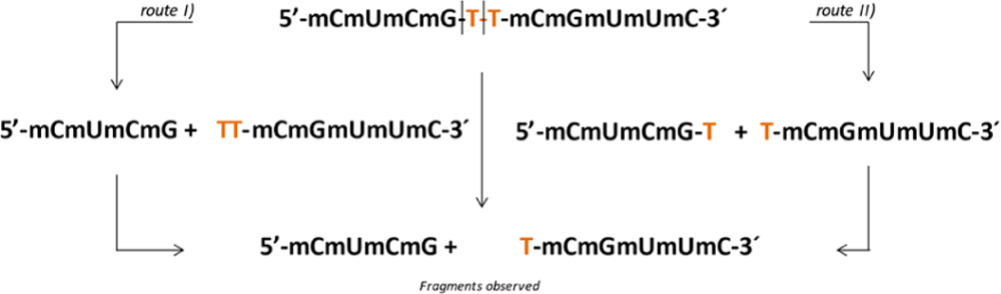
Alternative Cleavage Sequences Proposed for the Two-Step Hydrolysis of the Nude-dTdTprobe by MNase as Derived from the MALDI-TOF-MS Results

As proposed in Scheme 1, these results are compatible with two alternative mechanisms for double digestion of the probes by MNase, that is, either an initial hydrolysis of the **mGdT** phosphate bond followed by the cleavage of the key **dTdT nucleotide** pair in the **dTdTmCmGmUmUmC**-3′ fragment (route I) or, alternatively, an initial hydrolysis of the **dTdT** pair followed by the hydrolysis of the phosphate group linking together the **mGdT** pair (route II).

The actual route for the double cleavage mechanism was unequivocally identified to be both, in parallel, starting by route II and followed by route I and II (see Scheme 1).

### 3.3 Unraveling the Double Cleavage Mechanism

**Figure 5:**
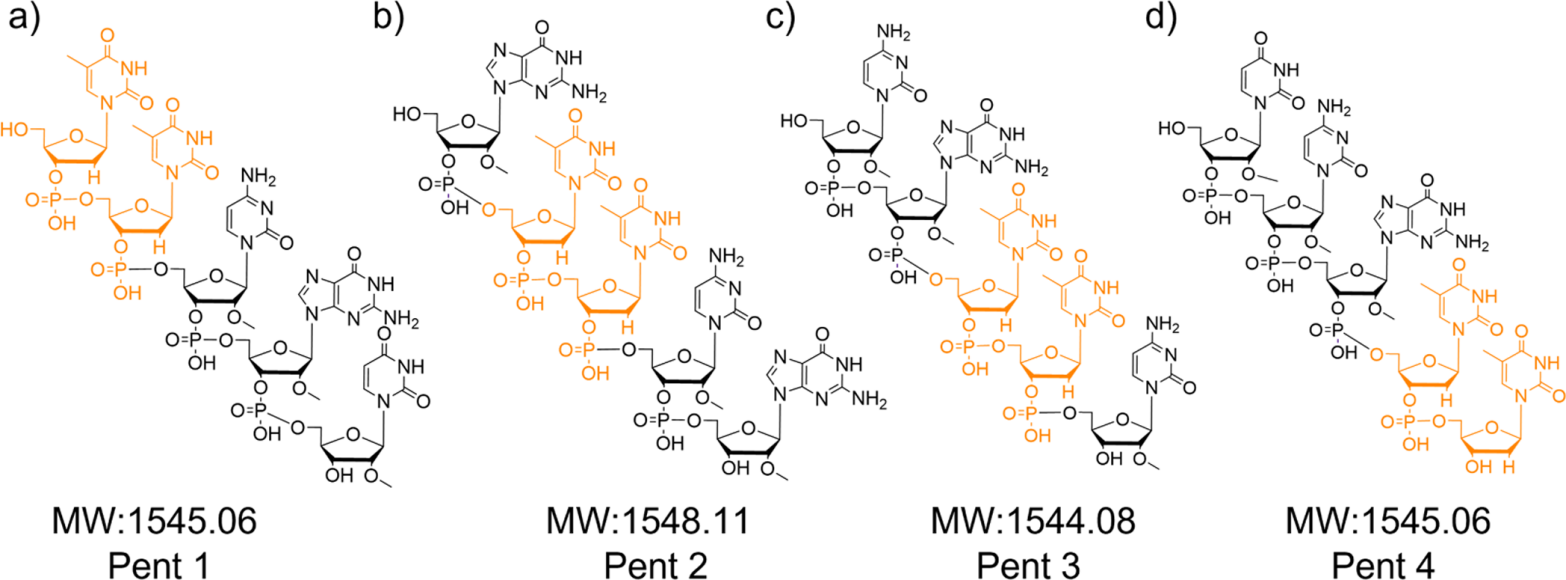
Chemical structure and molecular weight (MW, Da) of the four pentamers derived from the nude-dTdTprobe: (a) pent#1, (b) pent#2, (c) pent#3, and (d) pent#4.

The proposed double cleavage mechanism of the FRET-dTdTprobe was further investigated by means of virtual docking as well as UPLC experiments. Virtual docking experiments were performed with shortened molecular models of the nude-dTdTprobe, consisting of four pentameric oligonucleotide probes, pent#1 to pent#4, (see Figure 5, Table 2, and Supporting Information Figures S6 and S7 for additional details). The latter were tailored to address several important questions in the field of the design and development of oligonucleotide-based biosensors: (a) find the minimum substrate necessary to carry out the biosensing action with both specificity and efficiency; (b) improve the physicochemical properties that may favor a larger biodistribution in potential *in vivo* applications; and (c) decrease the entropy/degrees of freedom of the probe, which would simultaneously favor the receptor binding process/energetics in both experimental and virtual docking studies. Also, the performance of experiments with shortened sequences of the nude-dTdTprobe will benefit from the docking of ligands having less degrees of freedom.

**Table 2:** Sequences of the Pentameric Probes Tested

probe	sequence
pent#1	5′-**dT-dT-**mC-mG-mU-3′
pent#2	5′-mG-**dT-dT-**mC-mG-3′
pent#3	5′-mC-mG-**dT-dT-**mC-3′
pent#4	5′-mU-mC-mG-**dT-dT**-3′

The design of the pentamers consists of four truncated versions of the nude-dTdT parental probe, having dTdT at different positions, as follows: pent#1 with dTdT at positions 5–9; pent#2 at positions 4–8; pent#3 at positions 3–7; and pent#4 at positions 2–6. As discussed below, pent#3 and pent#4 were experimentally proven to serve as appropriate molecular models of the parental nude-dTdTprobe in terms of cleavage site identification, whereas pent#1 and pent#2 were not, being later characterized as oligonucleotides lacking the MNase recognition site.

**Figure 6:**
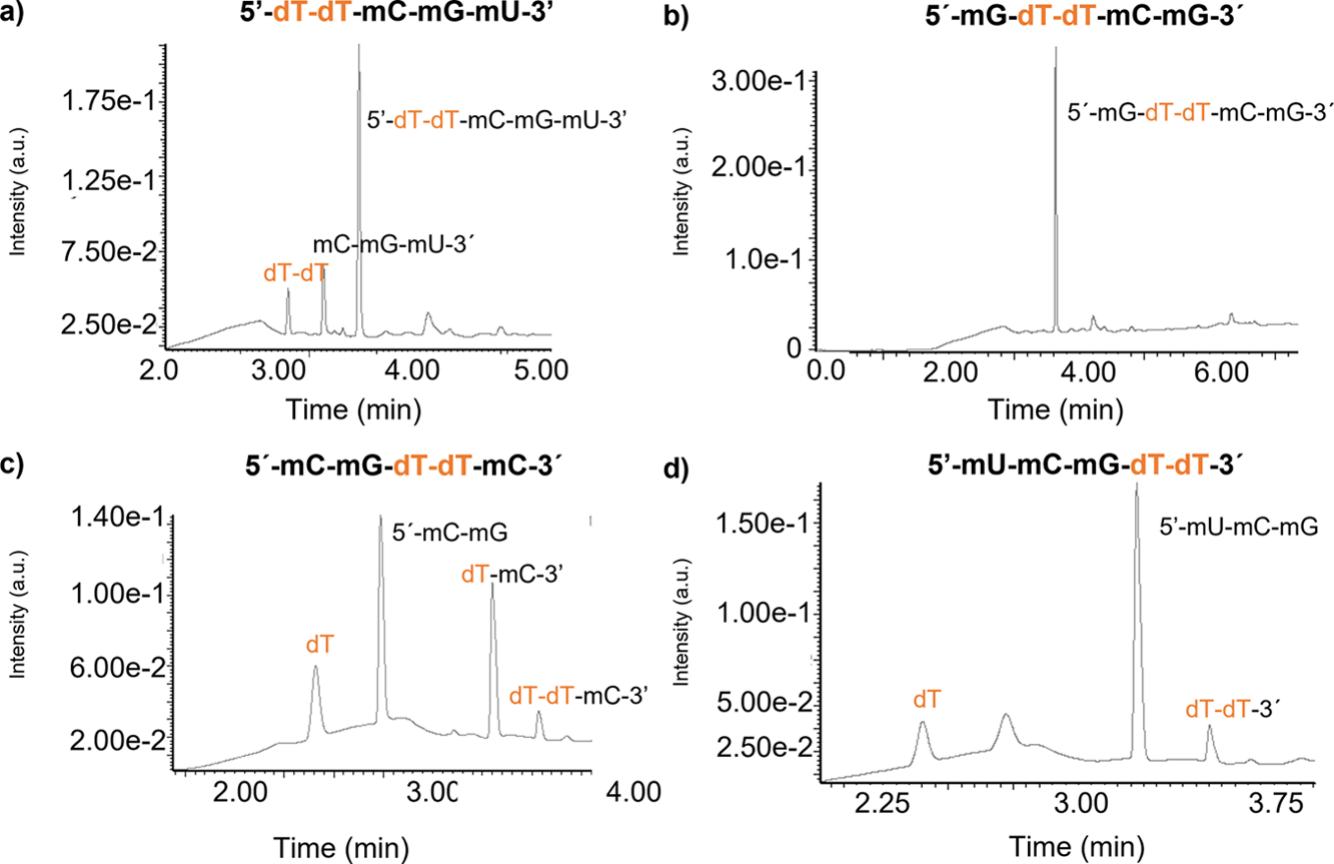
UPLC chromatogram of the four pentamers and digestion products after 1 h incubation time with MNase: (a) pent#1; (b) pent#2; (c) pent#3; and (d) pent#4 (see Figure S6). The dT nucleotide is colored orange in each probe’s digestion fragment.

The selection/identification of the most appropriate model of the nude- or FRET-dTdTprobe to be used with docking purposes was carried out by incubating the pentamers with *S. aureus* MNase and analyzing their digestion products by means of UPLC–MS (Figure 6). The latter experiments revealed, for instance, that pent#1 undergoes one single and yet poor phosphate bond hydrolysis under the reaction conditions used (Figure 6a). Briefly, pent#1 yielded small quantities of fragments **5′-dTdT** and **mC-mG-mU-3′,** yet the native nondigested pentamer was largely found on the analyzed digestion mixture, suggesting that the cleavage efficiency was relatively low. Interestingly, pent#2 was found to be completely resistant to digestion (Figure 6b), while pent#3 and #4 were largely digested by MNase (Figure 6c,d), and they both underwent two equivalent scissions, as did the original nude-dTdTprobe: cleavage at the 5′-side deoxythymidine position, yielding fragments 5′-**mC-mG** and **dT-dT-mC-**3′ in the case of pent#3, and subsequent hydrolysis of the **dTdT** phosphate bond, returning fragments **dT-mC-**3′ and **dT** [phosphate-bearing dT(PO_4_H_2_)]. Likewise, in the case of pent#4, the first cleavage yielded fragments 5′-**mU-mC-mG** and **dT-dT-**3′, while the latter fragment returned two deoxythymidine monomers after the second hydrolysis [dT and dT(PO_4_H_2_)].

Since these results were equivalent to those obtained with the native nude**-**dTdTprobe, pent#3 and pent#4 were identified as appropriate molecular models and therefore used in virtual docking experiments as substrates to be docked to the enzyme, which shed light into the binding mode and conformational properties of the catalytic complex. Pent#2 was also included in the virtual study as a negative control of the cleavage process.

**Figure 7:**
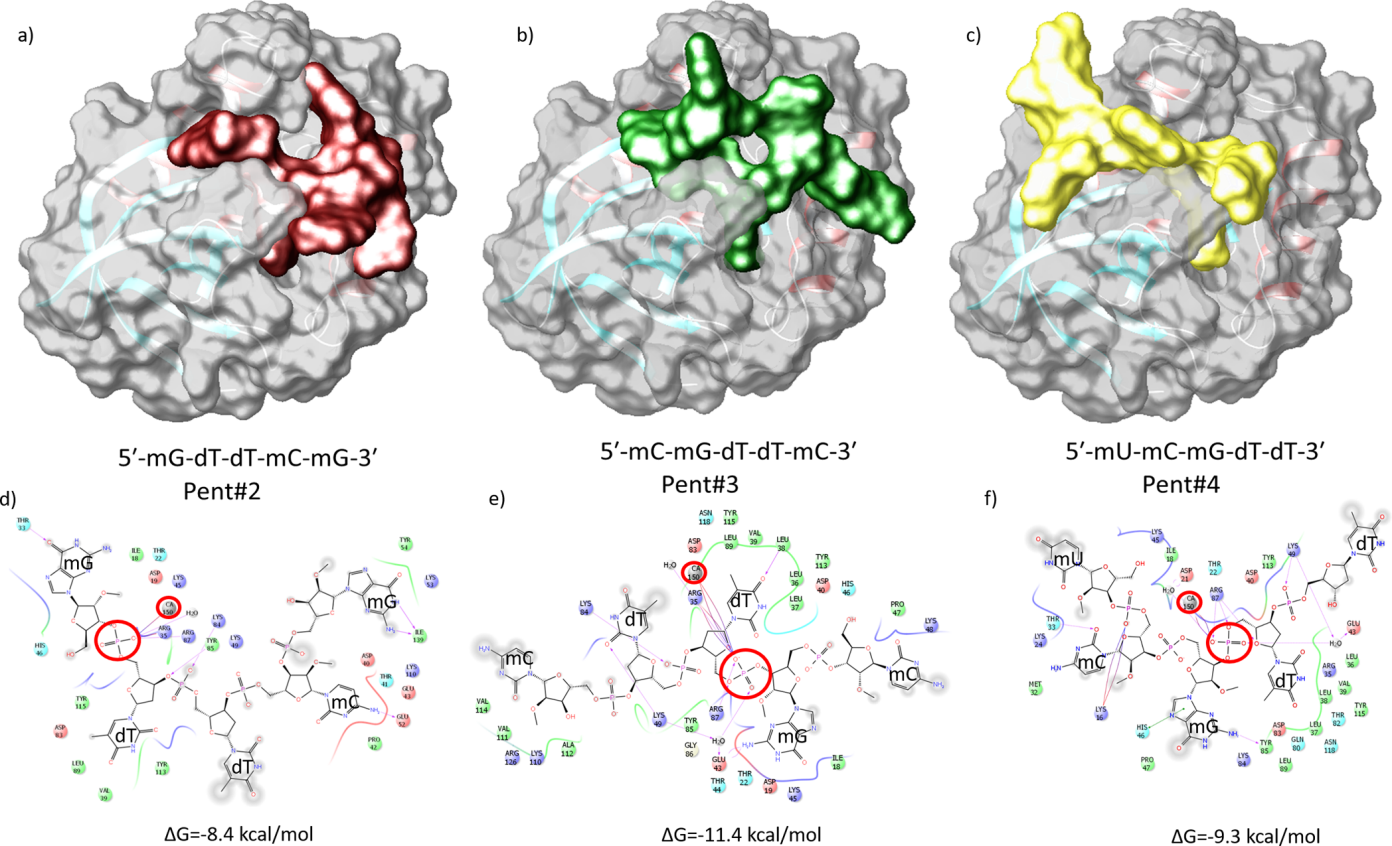
Surface representation (top) and ligand interaction diagram and Δ*G* binding energies (bottom), respectively, of catalytically nonrelevant poses of pent#2 (a,d) and catalytically relevant poses for pent#3 (b,e) and pent#4 (c,f). Red circles highlight the phosphate group coordinated to the calcium cation. The colors indicate the residue (or species) type: red—acidic (Asp and Glu); green—hydrophobic (Ala, Val, Ile, Leu, Tyr, Phe, Trp, Met, Cys, and Pro); purple—basic (Hip, Lys, and Arg); blue—polar (Ser, Thr, Gln, Asn, His, Hie, and Hid); light gray—other (Gly and water); and darker gray—metal atoms. Interactions with the protein are marked with lines between ligand atoms and protein residues: solid pink—H-bonds to the protein backbone; dotted pink—H-bonds to protein side chains; green—pi–pi stacking interactions; and orange—pi–cation interactions.

Thus, flexible docking of pent#2, pent#3, and pent#4 to the enzyme structure (see the Experimental Section and Supporting Information) was carried out, which generated a large variety of poses (snapshots of alternative binding modes of the ligand–receptor pair), derived from the large number of degrees of freedom still present in the pentameric probes. Poses were analyzed and classified as catalytically relevant or irrelevant depending on the presence or absence in the generated complex of certain key interactions between the probe and the enzyme, which were identified by Cotton et al. in previous reports on the phosphodiester bond hydrolysis mechanism (see the Experimental Section and Supporting Information, Figure S5).^16^ The application of these criteria allowed the identification of catalytically meaningful poses in the case of pent#3 and pent#4, but no meaningful poses were found for pent#2. Thus, Figure 7 shows the ligand interaction diagrams of the selected poses of pent#2, pent#3, and pent#4, where the phosphate group preceding the **dTdT** pair in pent#3 and pent#4 coordinates to the central cation, and simultaneously, all other relevant interactions with the key reported residues in the active site of the enzyme were also established. Interestingly, no catalytically relevant pose was identified for the case of pent#2; multiple poses of this pentamer showed phosphate group coordination to the central calcium cation, but they could not be considered as relevant due to the absence of other catalytically required interactions.

A closer look at the binding modes of pent#2, pent#3, and pent#4 to MNase revealed that the recognition of **mG** by means of its insertion in a pocket flanked by residues GLU43 and ASP19 may facilitate the coordination to the Ca^2+^ cation of the phosphate group preceding the **dTdT** couple (see Figures S4–S8 in the Supporting Information). The large affinity of the said pocket toward **mG** is also observed in pent#2, which is the only pentamer with two **mG** nucleotides and actually accommodates **5′-mG** in the identified pocket instead of **mG-3́**. The latter mG insertion promotes an alternative binding mode and conformation of the pentamer (as compared to pent#3 and pent#4), which translates into noncatalytically relevant poses. Therefore, the presence of the **mCmGdT** tandem in the pentamer (and the original dodecamer probe) seems to be crucial for the proper recognition and posterior hydrolysis of the **dTdT** phosphate bond; that is, the **mCmG** nucleotide pair sitting by the **dTdT** dimer is responsible for improving the cleavage performance and specificity of MNase, which implies that at least two chemically modified nucleotides are needed at the 5′-thymine cleavage site. Alternatively, poses obtained with pent#2, where the phosphate group between both **dT** nucleotides was coordinated to Ca^2+^, lacked some or all of the catalytically relevant interactions, and this pentamer was therefore not considered further as an appropriate molecular model of the nude-dTdTprobe. Even so, pent#2 was used as a negative control in the virtual docking experiments described below, that is, as a ligand that should not yield positive docking results.

**Figure 8:**
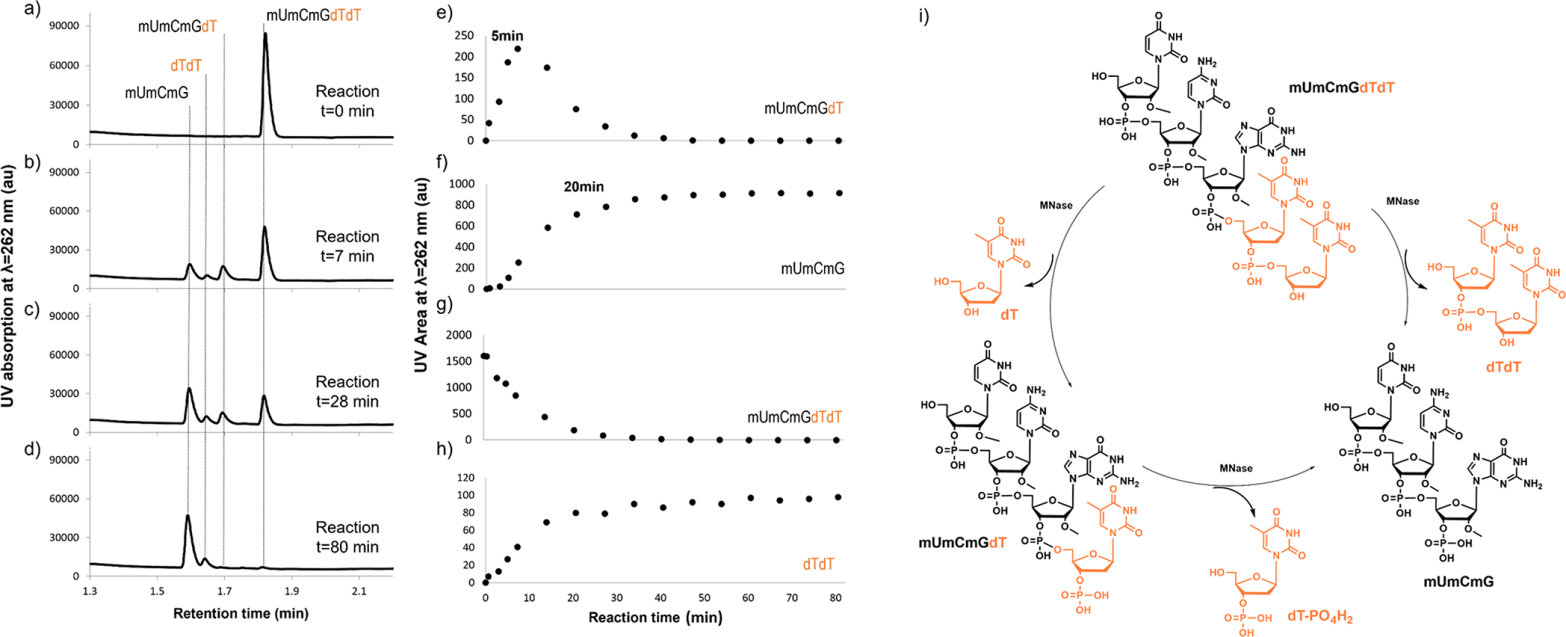
(a–d) UPLC chromatograms at four random reaction/incubation times and (e–h) time evolution of the area under the curve of the four peaks identified in the UPLC chromatograms of pent#4 (mUmCmGdTdT) as well as fragments generated upon incubation with MNase (see the Experimental Section for further details). (i) Catalytic cycle of the double hydrolysis caused by MNase on the pentameric oligonucleotide pent#4. dT nucleotide is colored orange in each probe’s digestion fragment.

To find out more information about the catalytic hydrolysis and elucidate important details of its catalytic order of action, UPLC kinetic experiments of the incubation of MNase (0.1 U/μL) with pent#4 (5 pmol/μL) were carried out in an 80 min reaction (Figure 8 and Supporting Information Table S2). A total of 16 UPLC chromatograms were acquired, derived from an injection volume of 10 μL every 6.66 min. UPLC chromatograms at four representative incubation times (*t* = 0, *t* = 7, *t* = 28, and *t* = 80 min) shown in Figure 8a–d revealed a double cleavage process started by pent#4, as concluded by the transient detection of fragment mUmCmGdT at *t* = 5 min (Figure 8e).

This intermediate is present during the first few minutes, which was completely digested by *t* = 20 min into fragments **mUmCmG** (Figure 8f) and 3′-phosphate deoxythymine **dT** (see a complete chromatogram in Figures S20–23). Simultaneously, the native pentamer (Figure 8g) was digested into fragments **mUmCmG** (Figure 8f) and the **dTdT** pair (Figure 8h).

The time evolution of four of the five fragments generated during the reaction (Figure 8e–h) allowed us to elucidate the catalytic cycle (see Figure 8i) of the reaction of *S. aureus* MNase on pent#4. This reaction implies a double hydrolysis mechanism, starting with **dTdT** pair hydrolysis in the first instance and followed by **mGdT** pair hydrolysis in the second (see Table S2).

Moreover, we have confirmed experimentally that FRET-pent#4 (fluorophore-labeled pent#4) maintains the detection capabilities of the parental FRET-dTdT probe (Figure S25). Importantly, we have also observed a significant improvement in the ARF of FRET-pent#4 [125.7 (±2.73)] over that of the parental FRET-dTdT probe [27.5 (±1.16)], indicating that more efficient fluorescence quenching was obtained by this truncated version (Figure S26).

**Figure 9:**
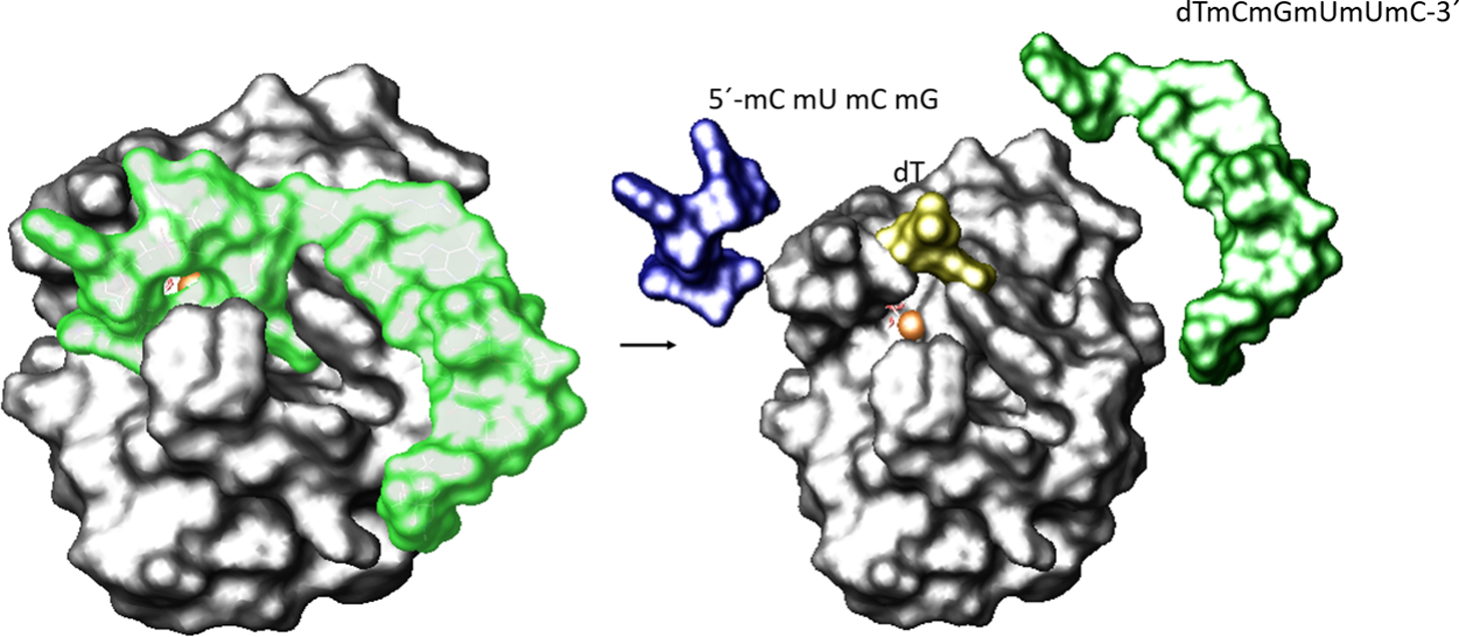
Left: Surface representation of the proposed molecular interaction between the parental nude-dTdTprobe and MNase of *S. aureus* before hydrolysis and Right: after hydrolysis of two subsequent phosphate groups.

Our findings are summarized in Figure 9, where general pictures of the nude-dTdTprobe in complex with MNase before and after hydrolysis are represented.

## 4 Conclusions

In summary, we confirm the specificity and selectivity of the dTdT nuclease activatable oligonucleotide probe to specifically detect *S. aureus* based on its MNase activity; also, we describe for the first time the full hydrolysis process undergone by the full-length probe, which implies double subsequent cleavages at the 5′-side position of both dT nucleotides, starting between the dTdT dimer and followed by the 5′-mG-dT cleavage. Appropriate molecular pentameric models of the nude-dTdTprobe and a fluorophore-labeled pentamer (FRET-pent#4) were designed and also validated experimentally, and their virtual binding mode was used to identify key catalytic interactions with the calcium cation, ARG35, ARG87, and a catalytic water molecule H-bonded to residue GLU43. Docking experiments pointed to a particular pair of modified nucleotides (mCmG) at the 5′-side of the nuclease’s cleavage site as necessary for probe recognition and binding. Specifically, the insertion of mG in a pocket flanked by residues GLU43 and ASP19 was found to be critical to trigger the subsequent hydrolysis of the probe at the active site pocket of MNase. These results represent an important step forward in the understanding of the mode of action, specificity, and efficiency of NAOPs in the context of nuclease activity detection and diagnosis, as well as the first description of the plausible truncation and reduction in size to a minimum of five nucleotides of an efficient oligonucleotide substrate.

## Supporting Information Available

Chemical structure of the FRET-dTdTprobe, details on FRET assays, MALDI-TOF experiments, phosphate bond hydrolysis mechanism, virtual docking experiments, UPLC experiment results, mass spectra, and details related to the catalytic cycle of the cleavage process proposed, pent#4’s FRET assays and activation ratio (PDF)
SMILES formula of the nude-dTdT probe and pentamers pent#1–pent#4(CSV)
Docking models of the binary complexes between 1SNC and pent#2 (PDB)
Docking models of the binary complexes between 1SNC and pent#3 (PDB)
Docking models of the binary complexes between 1SNC and pent#4 (PDB)

## Supplementary Material

jm1c00884_si_002.csv

jm1c00884_si_003.pdb

jm1c00884_si_004.pdb

jm1c00884_si_005.pdb

## Acronyms and Abbreviations

A: Agar
ATCC: American Type Culture Collection
a.u.: arbitrary units
dT: deoxythymidines
FAM: fluorescein amidite
FRET: fluorescence resonance energy transfer
MALDI-MS: matrix-assisted laser desorption/ionization mass spectrometry
UPLC-MS: ultra-performance liquid chromatography mass spectrometry
ESI-TOF: electrospray ionization time-of-flight
MALDI-TOF-MS: matrix-assisted laser desorption/ionization-time-of-flight mass spectrometry
MNase: micrococcal nuclease
NA: nutrient agar
NAA: nuclease activity assay
NAOPs: nuclease-activatable oligonucleotide probes
NB: nutrient broth
SAR: sequence/structure–activity relationship
*S. aureus*: *Staphylococcus aureus*
THB: Todd Hewitt broth
THP: thymidine-3′,5′-diphosphate
TQ2: tide quencher 2
UPLC-MS: ultra-performance liquid chromatography mass spectrometry
TSB: Trypticase soy broth
YE: yeast extract
XP: extra precision
