# Supplementary material for: Rational Design and Experimental Analysis of Short-Oligonucleotide Substrate Specificity for Targeting Bacterial Nucleases
Source: J Med Chem. 2021 Aug 30;64(17):12855–64. doi: 10.1021/acs.jmedchem.1c00884 (PMC8436206; doi:10.1021/acs.jmedchem.1c00884)
Supplement: Supplementary file 1 — jm1c00884_si_001.pdf [file jm1c00884_si_001.pdf]

SUPPORTING INFORMATION.

# Rational design and experimental analysis of short oligonucleotide substrate specificity for targeting bacterial nucleases.

Tania Jiménez,[a‡] Juliana Botero,[a,b,‡] Dorleta Otaegui,[c] Javier Calvo,[c] Frank J. Hernandez,[d,e*] Eider San Sebastian[b,*]

[a] Somaprobes S.L, Mikeletegi Pasealekua, 83, 20009 Donostia, Gipuzkoa
[b] Applied Chemistry Department, University of the Basque Country (UPV/EHU), 20018 San Sebastian, Spain
[c] Center for Cooperative Research in Biomaterials (CIC biomaGUNE), Basque Research and Technology Alliance (BRTA), San Sebastian 20014, Spain.
[d] Wallenberg Center for Molecular Medicine (WCMM), 58185 Linköping, Sweden
[e] Department of Physics, Chemistry and Biology, Linköping University, 58185 Linköping, Sweden
‡ These two authors contributed equally

Corresponding authors:
Eider San Sebastian –email: eider.sansebastian@ehu.eus
Frank J. Hernandez –email: frank.hernandez@liu.se

**S1. NAOPs.**
- **NAOPs data sheet.**
- **Chemical structure of NAOP (FRET-dTdT probe).**

**S2. FRET ASSAYS.**
- **NAOPs tested for specificity against five pathogen strains.**
- **Raw fluorescence intensity data.**

**S3. MALDI-TOF EXPERIMENTS**

**S4. PHOSPHATE BOND HYDROLYSIS**

**S5. VIRTUAL DOCKING EXPERIMENTS**

- **Identifications of key interactions between the substrate and key residues in the 1SNC nuclease.**
- **Virtual preparation of the MC nuclease.**
- **Ligand interaction diagram for THP docked to MC nuclease.**
- **Results of Pent#1, Pent#2, Pent#3 and Pent#4 docking to the prepared MC nuclease 1SNC.**

**S6. UPLC EXPERIMENTS, MASS SPECTRA AND CATALYTIC CYCLE OF THE CLEAVAGE PROCESS**

- **UPLC chromatogram of the four pentamers.**
- **Catalytic cycle of the digestion of pentamer 4 (pent#4) upon incubation with MNase. (t=14 min) and the corresponding Mass spectra.**
- **All chromatograms (t=0 – t=88 min)**
- **Kinetics study of the hydrolysis process**

**S7. FRET ASSAYS WITH FLUOROPHORE-LABELLED PENTAMER 4 (FRET-Pent#4)**

## S1. NAOPs

***NAOPs data sheet.***

All NAOPs were purchased at Biomers.net (Germany).

DNA, poly A, poly T, poly AT, 2´Ome, 2´F, FRET-dTdT and FRET-pent#4 probes are synthesized with fluorescein amidite (FAM) fluorophore at the 5'-end and the tide quencher 2 (TQ2) at the 3'-end. For this synthesis, a standard method of solid-phase phosphoramidite chemistry was used by the company provider, followed by high-performance liquid chromatography (HPLC) purification process. The probe identities were confirmed with matrix-assisted laser desorption ionization mass spectrometry (MALDI-MS). In all cases, the purity of the probes assessed with HPLC analysis, was typically greater than 95%. As a proof of purchase, here we report the supplier data sheets of all probes used in this study:

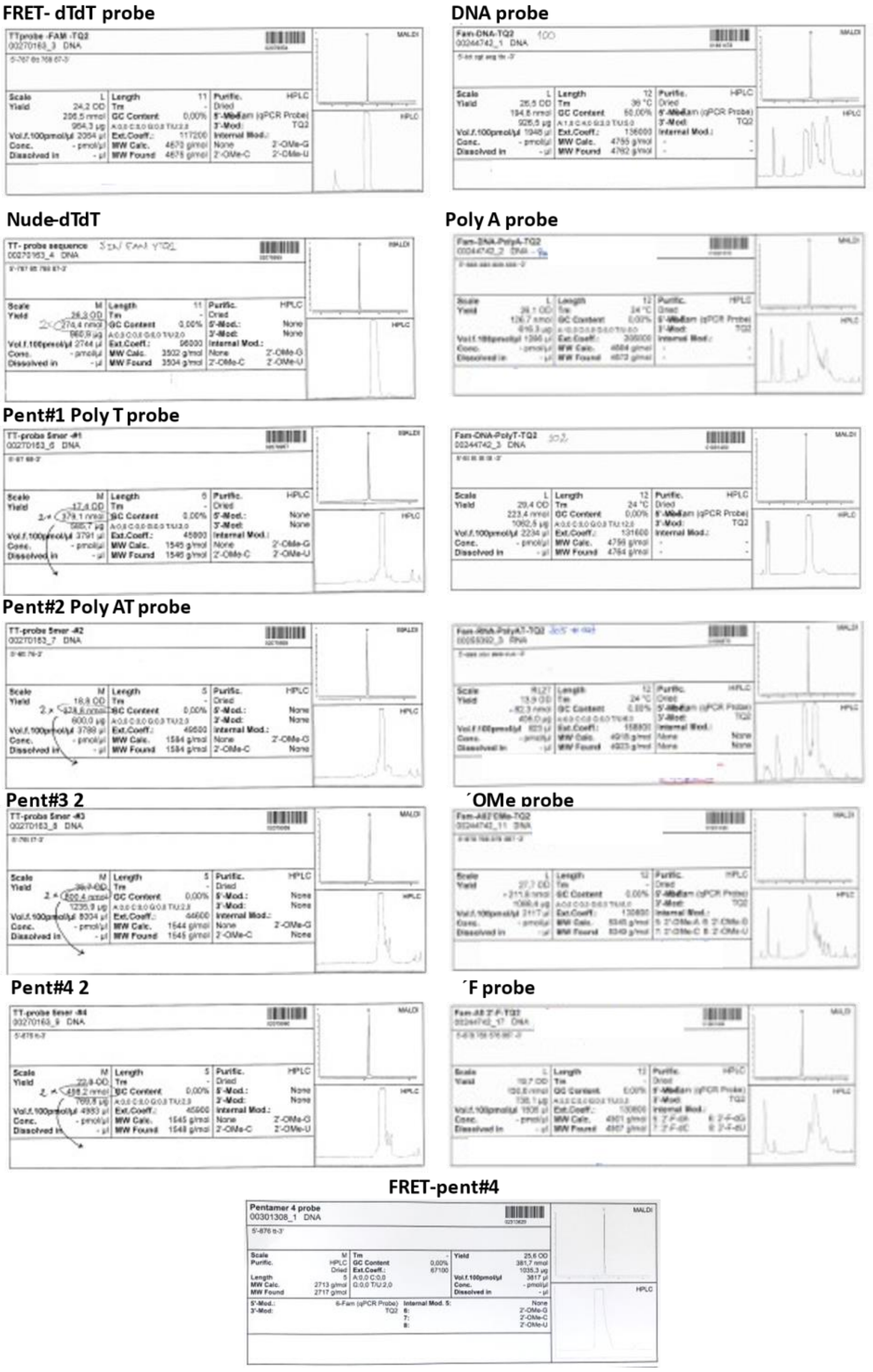

***Chemical structure of FRET-dTdT***

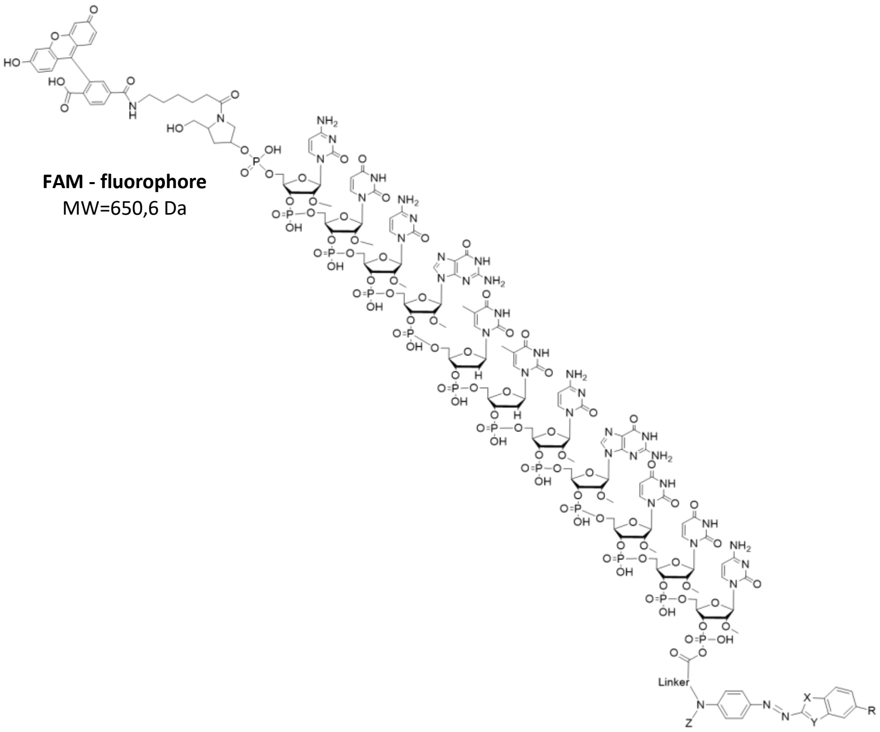


**FAM - fluorophore**
MW=650,6 Da

**TQ2 quencher**
MW=517 Da

**Figure S1.** Chemical structure of the FRET-dTdT probe (MW= 4673 Da)

## S2. FRET ASSAYS.

***NAOPs tested for specificity against five pathogen strains.***

**Table S1.** Oligonucleotide sequence of the 7 NAOPs tested against different bacterial strains.

| Name | Sequence |
|---|---|
| **DNA** | **5´-FAM-dTdCdTdCdGdTdAdCdGdTdTdC-3´-TQ2** |
| **Poly A** | **5´-FAM-dAdAdAdAdAdAdAdAdAdAdAdA-3´-TQ2** |
| **Poly T** | **5´-FAM-dTdTdTdTdTdTdTdTdTdTdTdT-3´-TQ2** |
| **Poly AT** | **5´-FAM-dAdAdAdTdTdTdAdAdAdTdTdT--3´-TQ2** |
| **2´OMe** | **5´-FAM-mUmCmUmCmGmUmAmCmGmUmUmC-3´-TQ2** |
| **2´F** | **5´-FAM- fUfCfUfCfGfUfAfCfGfUfUfC-3´-TQ2** |
| **FRET-dTdT** | **5´-FAM-mCmUmCmGdTdTmCmGmUmUmC-3´-TQ2** |

***Raw fluorescence intensity data***

**Table S1.** Raw fluorescence intensity data obtained upon incubating the seven NAOPs tested against different bacterial strains (±standard deviation).

| *probe* | *Fluorescence intensity (a.u)* | *S. dev. (±)* |
|---|---|---|
| **Control Buffer** | | |
| **DNA** | **29** | ***1.0*** |
| **PolyA** | **120** | ***3.2*** |
| **PolyT** | **40** | ***0.6*** |
| **PolyAT** | **16** | ***0.0*** |
| **2'Ome** | **11** | ***1.0*** |
| **2'F** | **12** | ***0.6*** |
| **dTdT** | **22** | ***1.2*** |
| **MNase (0.1U/μL)** | | |
| **DNA** | **650** | ***2.9*** |
| **PolyA** | **741** | ***4.0*** |
| **PolyT** | **696** | ***0.6*** |
| **PolyAT** | **609** | ***8.7*** |
| **2'Ome** | **18** | ***0.6*** |
| **2'F** | **44** | ***0.6*** |
| **dTdT** | **664** | ***11.4*** |
| ***Staphylococcus aureus*** **ATCC 29213** | | |
| **DNA** | **547** | ***14.9*** |
| **PolyA** | **725** | ***2.5*** |
| **PolyT** | **567** | ***15.7*** |
| **PolyAT** | **516** | ***10.6*** |
| **2'Ome** | **12** | ***0.6*** |
| **2'F** | **82** | ***2.3*** |
| **dTdT** | **504** | ***14.2*** |
| ***Proteus mirabilis*** **ATCC 25933** | | |
| **DNA** | **208** | ***3.5*** |
| **PolyA** | **431** | ***8.0*** |
| **PolyT** | **485** | ***12.1*** |
| **PolyAT** | **284** | ***2.6*** |
| **2'Ome** | **11** | ***0.6*** |
| **2'F** | **61** | ***2.6*** |
| **TT** | **57** | ***2.5*** |

| *probe* | *Fluorescence intensity (a.u)* | *S. dev.* |
|---|---|---|
| ***Klebsiella pneumoniae*** **ATCC 13883** | | |
| **DNA** | **34** | ***1.5*** |
| **PolyA** | **119** | ***4.5*** |
| **PolyT** | **47** | ***0.6*** |
| **PolyAT** | **36** | ***1.5*** |
| **2'Ome** | **9** | ***0.6*** |
| **2'F** | **14** | ***0.6*** |
| **TT** | **22** | ***1.0*** |
| ***Pseudomonas aeruginosa*** **ATCC 10145** | | |
| **DNA** | **29** | ***0.6*** |
| **PolyA** | **119** | ***4.6*** |
| **PolyT** | **46** | ***1.0*** |
| **PolyAT** | **21** | ***0.6*** |
| **2'Ome** | **11** | ***0.0*** |
| **2'F** | **11** | ***1.2*** |
| **TT** | **24** | ***0.6*** |
| ***Streptococcus pneumoniae*** **ATCC 49619** | | |
| **DNA** | **34** | ***1.2*** |
| **PolyA** | **115** | ***2.0*** |
| **PolyT** | **48** | ***0.0*** |
| **PolyAT** | **23** | ***0.6*** |
| **2'Ome** | **18** | ***1.0*** |
| **2'F** | **18** | ***1.2*** |
| **TT** | **29** | ***1.0*** |

## S3. MALDI-TOF EXPERIMENTS

Upon incubation of the FRET-dTdT probe (MW: 4673 Dalton) with the MNase, digestion fragments were analysed by means of MALDI-TOF experiments. As observed in the MALDI-TOF spectrum in Figure S2, the starting construct of 4673Dalton is cleavage into two detectable fragments (m/z: 1987 and 2399) after digestion of the FRET-dTdT probe with MNase. Note that the 5´-> 3´-nuclease activity of MNase promotes the cleavage of phosphodiester groups generating the 3´-hydrolyzed phosphate ends and the protonated 5'-oxygen atoms in the leaving fragment. The peaks detected in the MALDI-TOF spectrum correspond to cleavage products with masses compatible with fragments FAM-5'-mCmUmCmG and dTmCmGmUmUmC-3'-TQ2, which imply that the cleavage occurs at the 5' thymine's position. Signals corresponding to digestion fragments of sequences FAM-5'-mCmUmCmGdT or dTdTmCmGmUmUmC-3´-TQ2 were not observed under these reaction conditions. Thus, a third, low-mass and non-detectable fragment corresponding to dT-(3´-$PO_4H_2$) nucleotide of expected mass 322.21 Dalton is expected to be a product of the nuclease activity on the FRET-dTdT probe as well.

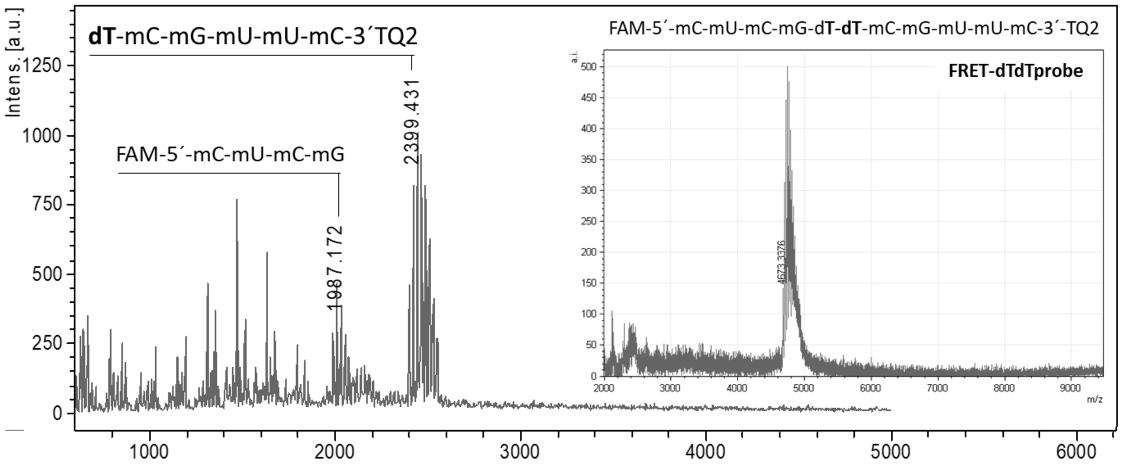


**Figure S2.** MALDI-TOF-MS spectrum of the digested and non-digested (inset) FRET-dTdT probe. According to the data sheet provided by Biomers Ltd, for FRET-dTdT probe: Calculated MW: 4670 g/mol. (Found MW: 4674 g/mol)

## S4. PHOSPHATE BOND HYDROLYSIS

***Proposed mechanism for the hydrolysis of a phosphate bond in a calcium dependent manner.***

According to Cotton et al. proceeds via the by cleavage of the 5'-P-O bond to yield a free 5'-hydroxyl group and a terminal 3'-phosphate monoester group (Figure S3). The latter description of the most plausible cleavage mechanism was derived from a careful analysis of the structure of the active site of the enzyme based on their interpretation of the nuclease-thymidine 3',5'-bisphosphate (pdTp)-$Ca^{2+}$ complex at 1.5-Å resolution and a certain known chemical and enzymological properties of the nuclease.

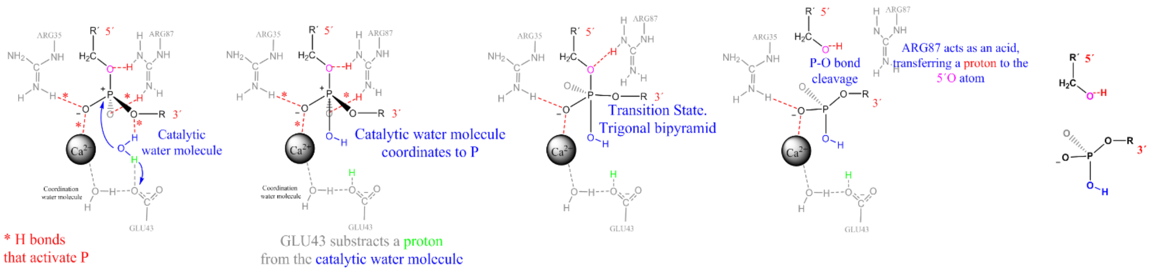


1 2 3 4

**Figure S3.** Schematic representation of the interactions that may be occurring in the catalytic complex formed between a phosphate group and key residues in the S. aureus MNase active site.

## S5. VIRTUAL DOCKING EXPERIMENTS

Virtual docking of several nucleotide probes to the MNase receptor structure was made with the Glide software package of Schrodinger inc. A careful analysis (beyond the scope of this report) of the wide variety of structures deposited in the PDB for this nuclease pointed to PDB ID= 1SNC[1] as the most appropriate to perform the virtual docking study. The latter crystal structure consists of the ternary complex of residues 7-141 of staphylococcal nuclease, $Ca^{2+}$, and the inhibitor thymidine-3',5'-diphosphate (THP) refined at 1.65 Å.

***Identifications of key interactions between the substrate and key residues in the MNase.***

The coordination sphere of the $Ca^{2+}$ cation found on the crystal structure of *S. aureus* MNase (PDB ID= 1SNC) consists of a heptacoordinated system (Figure S4), where three water molecules (HOH178, HOH187 and HOH195), residues ASP21, ASP40 and THR41 and the 5´ terminal phosphate group of THP ligand, coordinate to the central metal cation via oxygen atoms, giving rise to a MO7 coordination sphere. As opposed to ASP21 and ASP40 that coordinate via carboxylic sidechains, THR41 binds to metal via backbone amide oxygen atom.

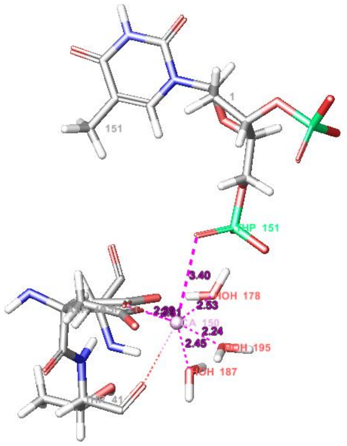


**Figure S4.** Details of the coordination sphere of the Ca2+ cation found in the active site of MNase (PDB ID=1SNC). Colour code: Carbon: grey; oxygen: red; hydrogen: white; nitrogen: blue; phosphorus: green; calcium: pink.

A deep analysis of the crystal structure of 1SNC reveals the presence of eight key interactions between the phosphate group of the THP substrate and various residues of the active site of the MNase:

a. Phosphate oxygen atom O1 establishes two H-bonds with two distinct water molecules (blue dots), which simultaneously establishes a second H-bond with a carboxylic oxygen of GLU 43 (orange dot).
b. Phosphate oxygen atom O2 establishes two H-bonds (green dots), both with a guanidinic H of the guanidinium moiety of ARG87 and ARG 35, respectively.
c. Phosphate oxygen atom O3 establishes three key interactions: an H-bond with a guanidinic H of the guanidinium moiety of ARG35 (grey dot), a second H-bond with one water molecule (red dot) and the coordination to the $Ca^{2+}$ atom.
d. Phosphate oxygen atom O4 establishes a single interaction with a guanidinic H of the guanidinium moiety of ARG87.

The same interactions were described by Cotton *et al*. when they reported[2] the structure of the staphylococcal nuclease (EC 3.1.4.7)-thymidine 3',5'-bisphosphate-$Ca^{2+}$ (enzyme-inhibitor) complex, except that phosphate oxygen atom O1 establishes a single H-bond with a water molecule.

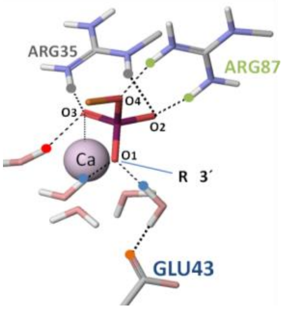


**Figure S5**. Left: THP substrate. Right: detail of the interactions established between THP and key residues found in the active site of MCNase (PDB ID=1SNC). Only key sidechain atoms are shown for Arg35, Arg87 and Glu43, while THP was reduced to the (R´)-$CH_2$-phosphate group. Colour code: Carbon: grey; oxygen: red; hydrogen: white; nitrogen: blue; phosphorus: violet; calcium: pink.

***Virtual preparation of the MNase.***

The crystal structure of any receptor deposited in the PDB needs a delicate “preparation” process prior to be used in any virtual docking study. The “preparation” steps may include the addition of missing hydrogens, missing side-chains and/or loops, assigning bond orders, creating zero-order bond to metals, and optimization of H-bond networks, among others. The quality of the “prepared” receptor structure generated in this study was confirmed upon comparison of the binding mode of THP to MNase in 1SNC (PDB ID= 1SNC) and the binding mode of THP docked to the “prepared” structure of the enzyme (Figure S6). The small RMSD value (0,51 Å) between both THP conformations validate the structure of the “prepared” nuclease receptor for further docking studies.

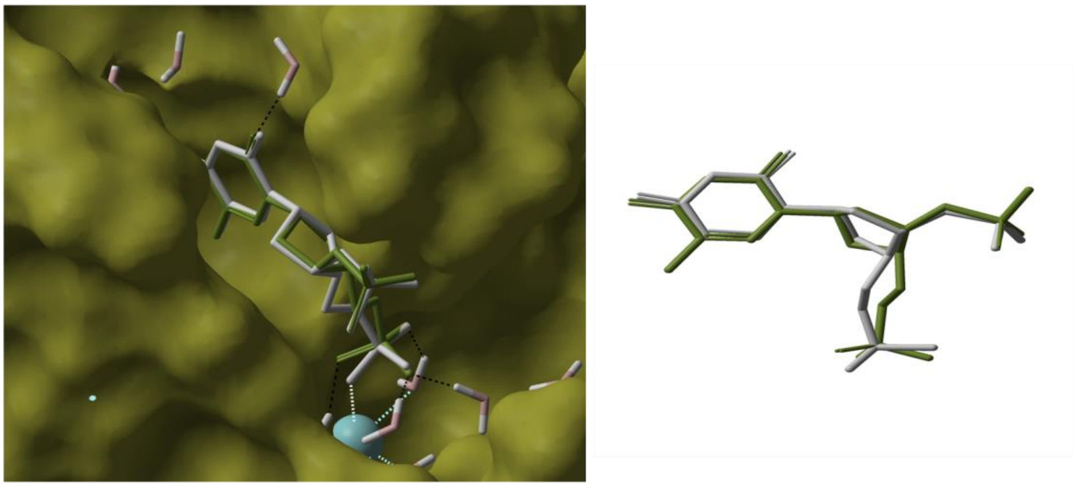

**Figure S6.** Left: Detail of the active site of the THP-MCNase complex as found in the crystal structure (white) and the one obtained upon a virtual docking of the substrate to the “prepared” receptor. Right: View of the overlaid structures (RMSD 0,51 Å) of THP in both complexes.

***Ligand interaction diagram for THP docked to MC nuclease.***

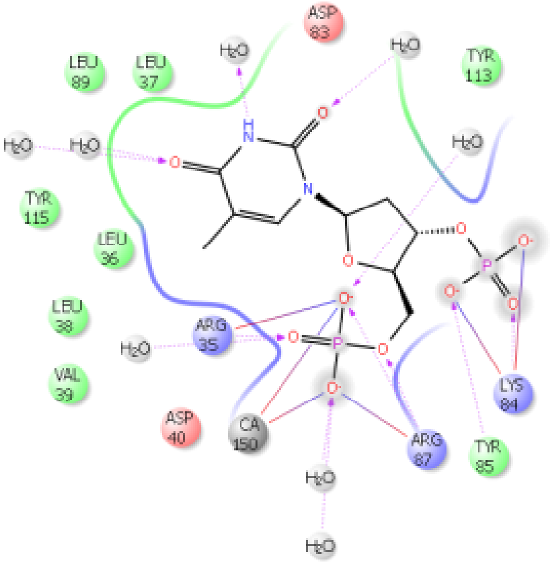


**Figure S7.** Ligand interaction diagram for THP in the MNase structure with PDB ID 1SNC, displaying key interactions with neighbouring residues. The colors indicate the residue (or species) type: red—acidic (Asp, Glu); green—hydrophobic (Ala, Val, Ile, Leu, Tyr, Phe, Trp, Met, Cys, Pro); purple—basic (Hip, Lys, Arg); blue—polar (Ser, Thr, Gln, Asn, His, Hie, Hid); light gray—other (Gly, water); darker gray—metal atoms. Interactions with the protein are marked with lines between ligand atoms and protein residues: Solid pink—H-bonds to the protein backbone; Dotted pink—H-bonds to protein side chains; Green—pi-pi stacking interactions; Orange—pi-cation interactions.

***Results of Pent#1, Pent#2, Pent#3 and Pent#4 docking to the prepared MC nuclease 1SNC***

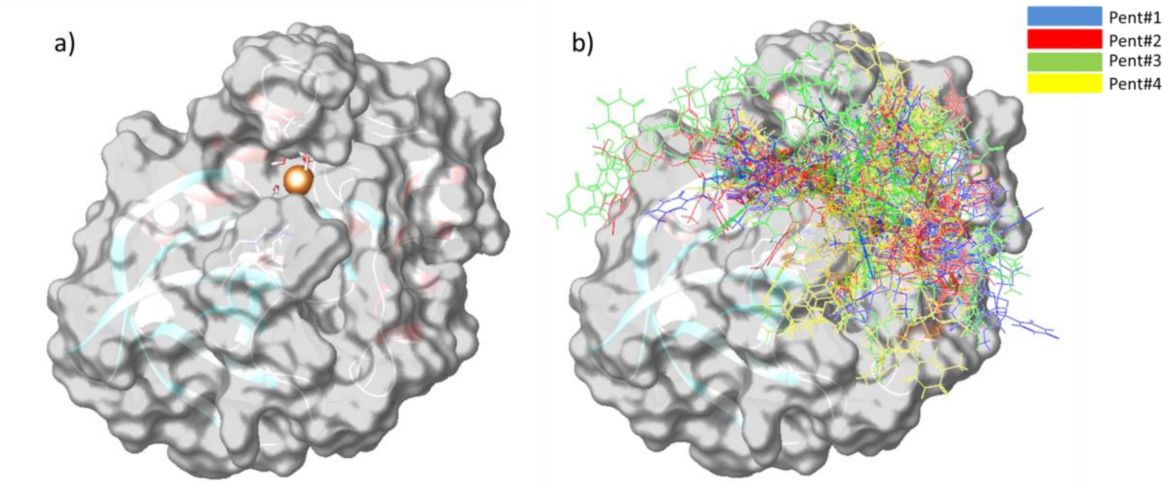


**Figure S8.** (a) Surface representation of MNase and its active site where the calcium cation (orange sphere) and the catalytic water molecules are shown. (b) Representation of multiple poses, either catalytically relevant or irrelevant, obtained upon flexible docking for Pent#1 (blue), Pent#2 (red), Pent#3 (green) and Pent#4 (yellow) to MNase.

## S6. UPLC EXPERIMENTS, MASS SPECTRA AND CATALYTIC CYCLE OF THE CLEAVAGE PROCESS

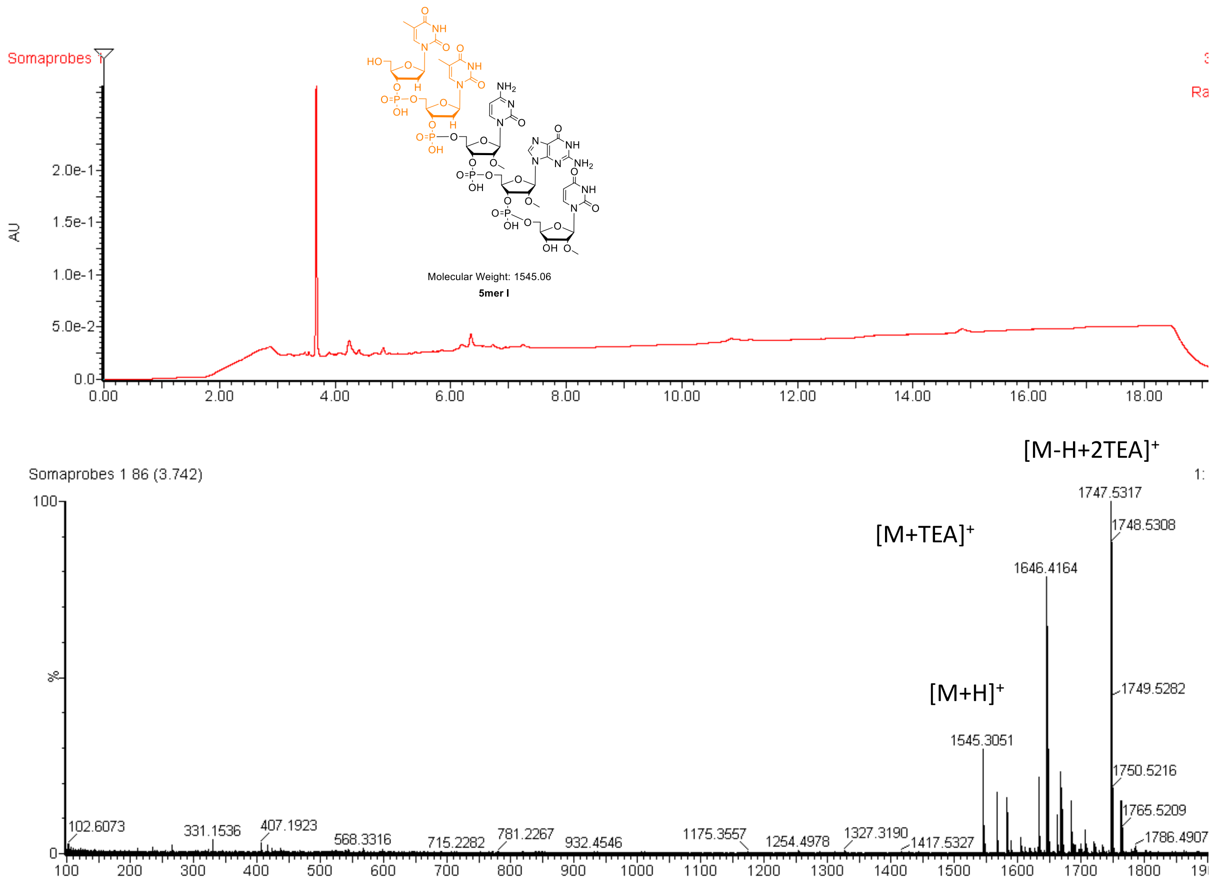


**Figure S9.** UPLC chromatogram (top) and mass spectrum (bottom) of pent#1 (λ=262 nm).

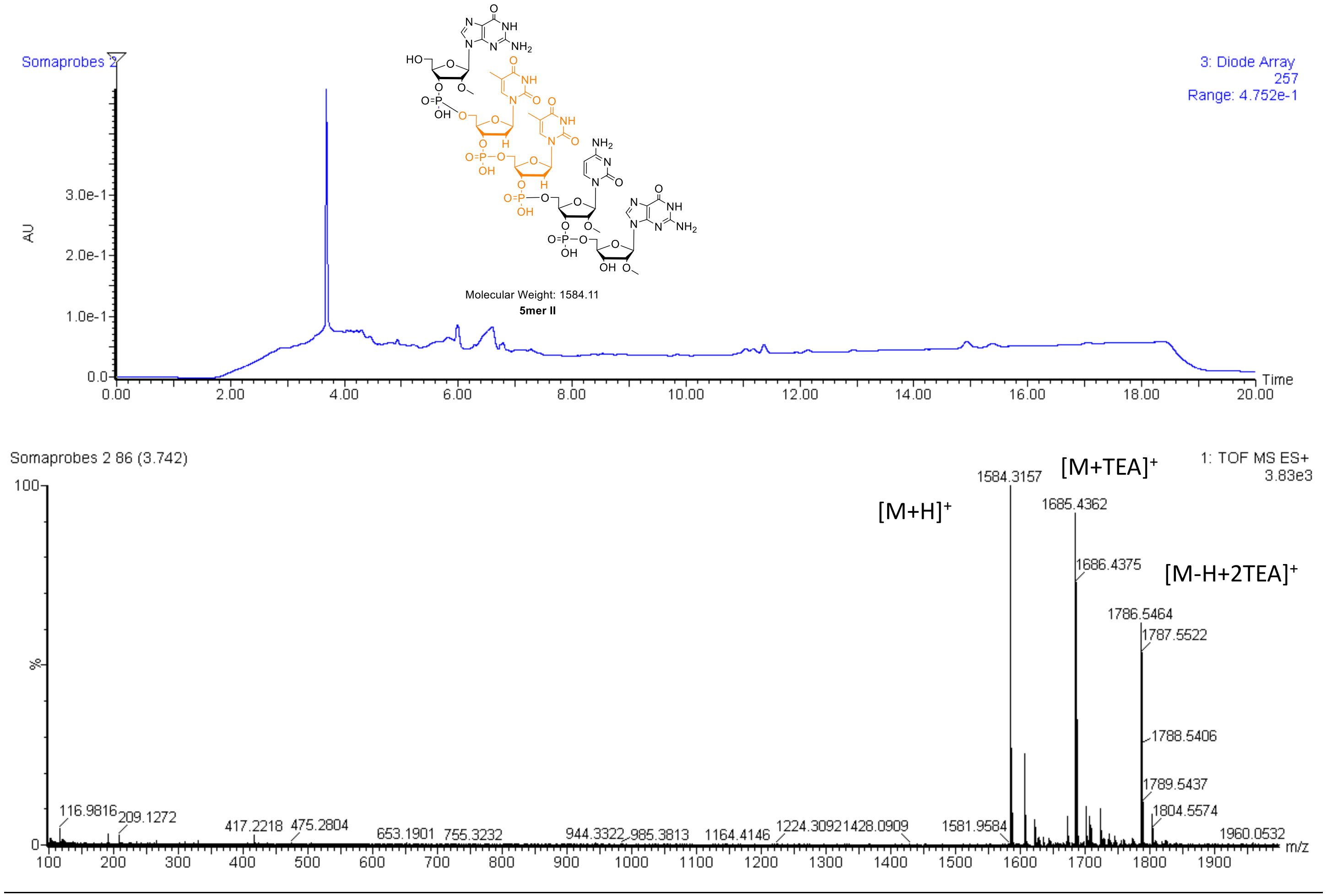


**Figure S10.** UPLC chromatogram (top) and mass spectrum (bottom) of pent#2 (λ=262 nm).

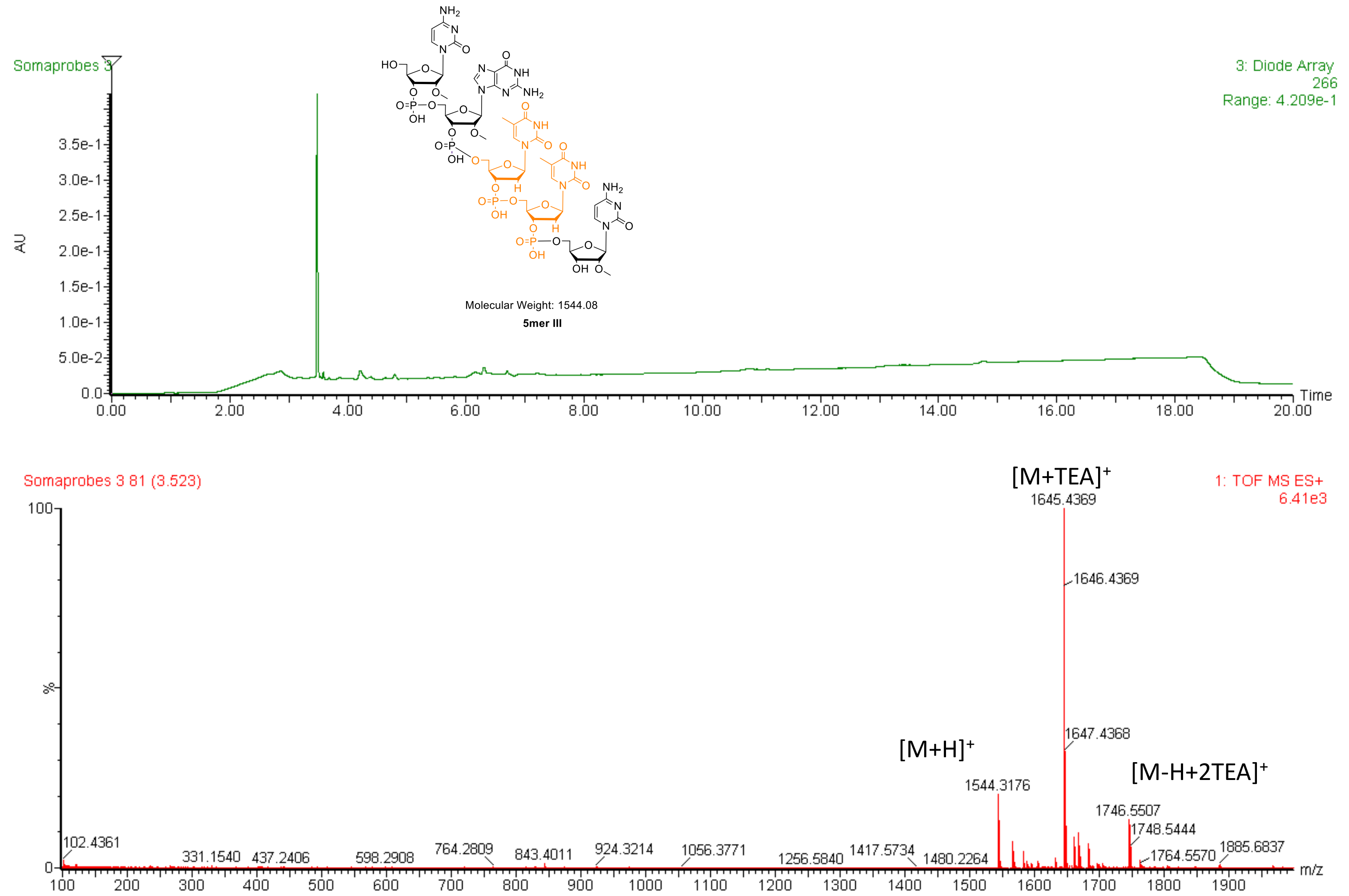


**Figure S11.** UPLC chromatogram (top) and mass spectrum (bottom) of pent#3 (λ=262 nm).

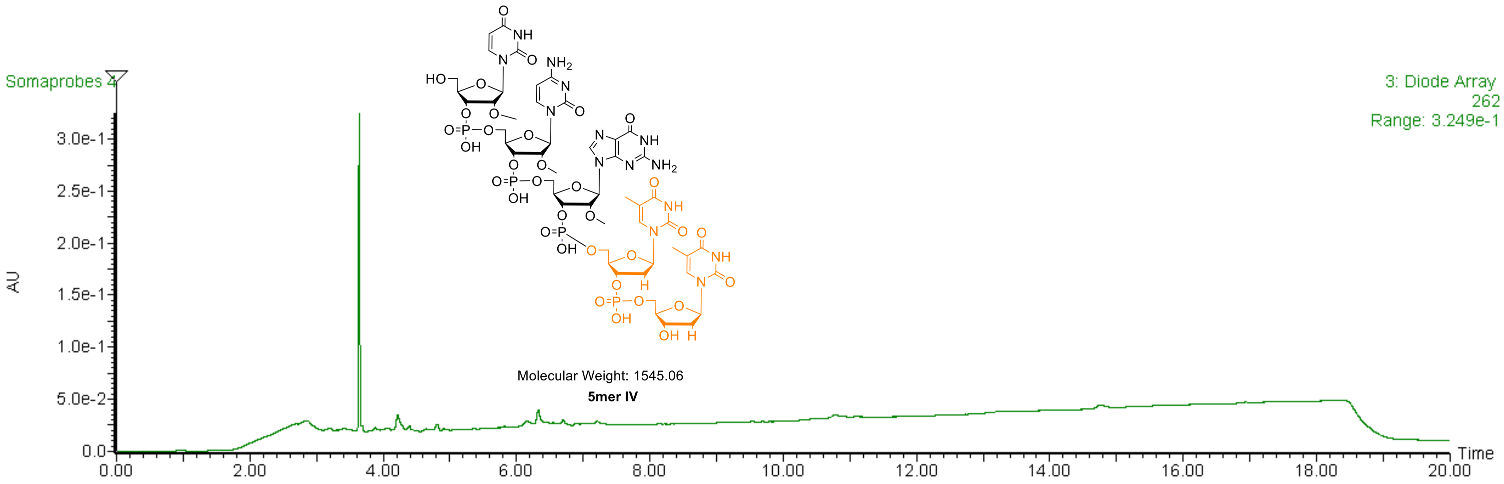


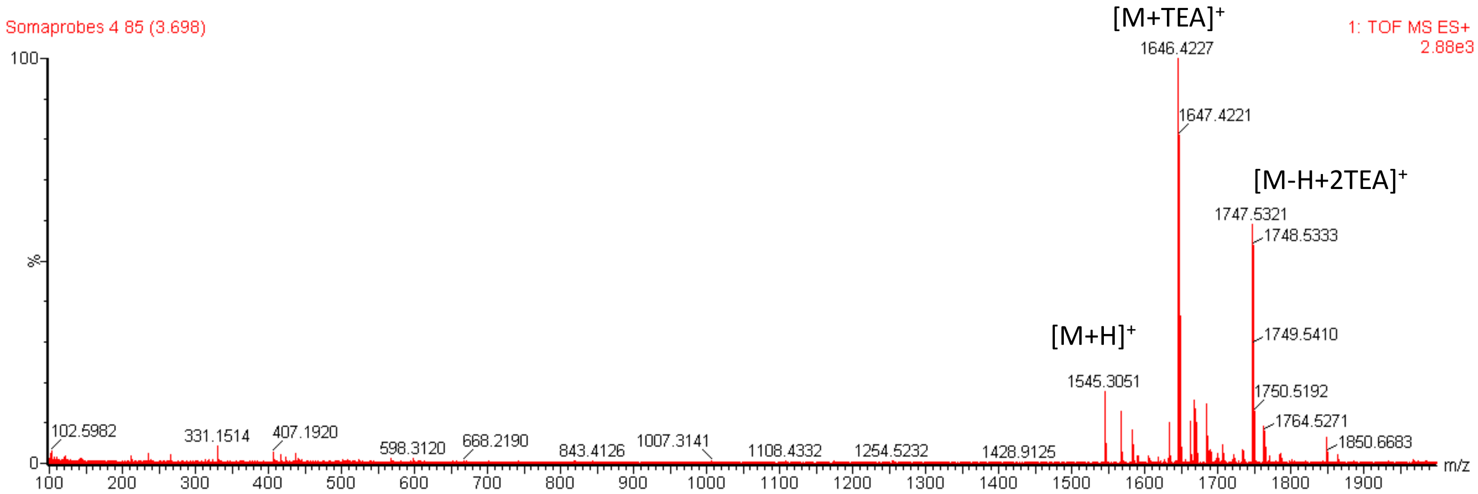


**Figure S12.** UPLC chromatogram (top) and mass spectrum (bottom) of pent#4 (λ=262 nm).

***UPLC chromatogram of the digestion fragments of the four pentamers after 1 h incubation with MNase.***

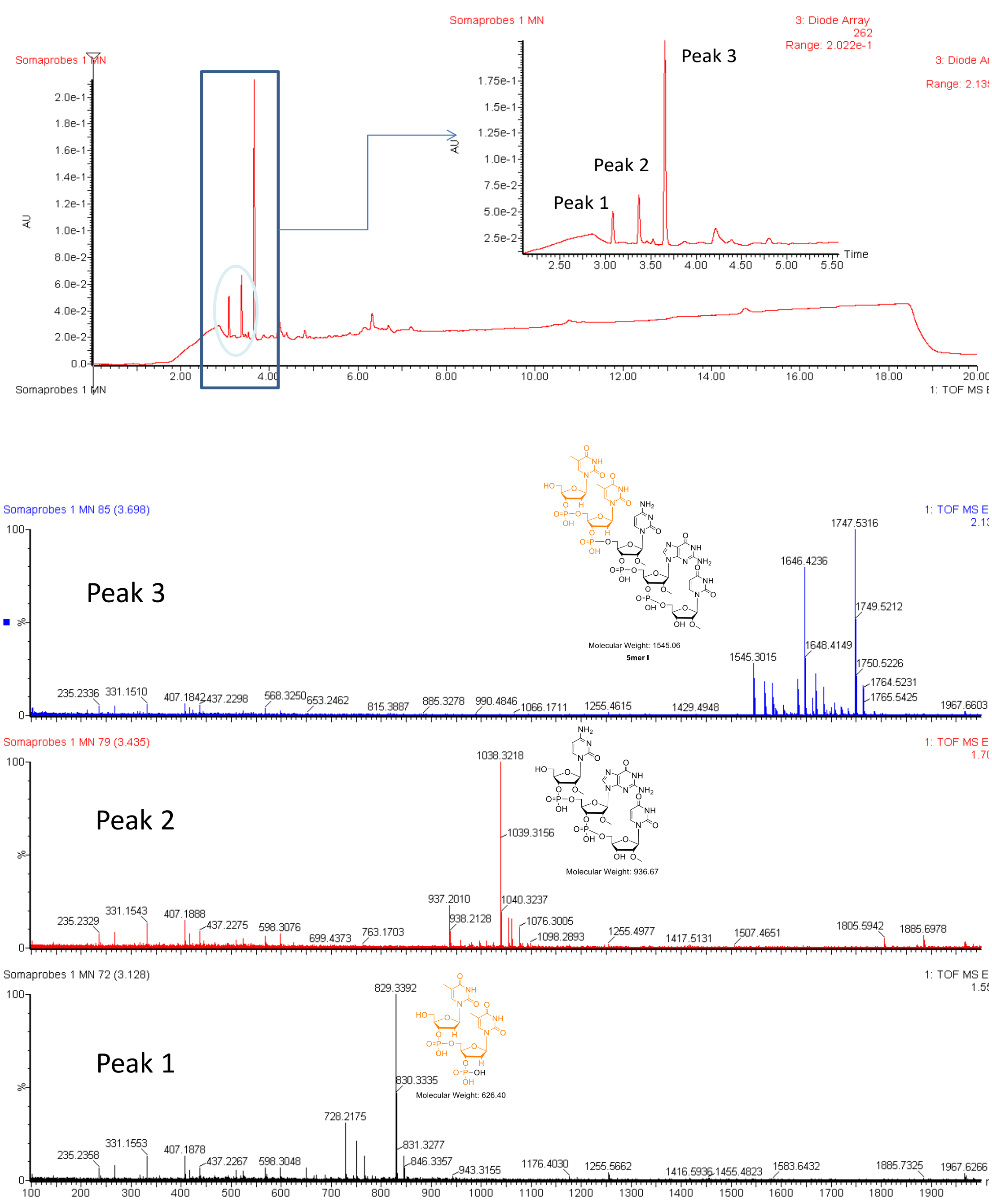


**Figure S13.** UPLC chromatogram (top, λ=262 nm) and mass spectra (bottom) of the digestion fragments of pent#1 upon 1h incubation with MNase.

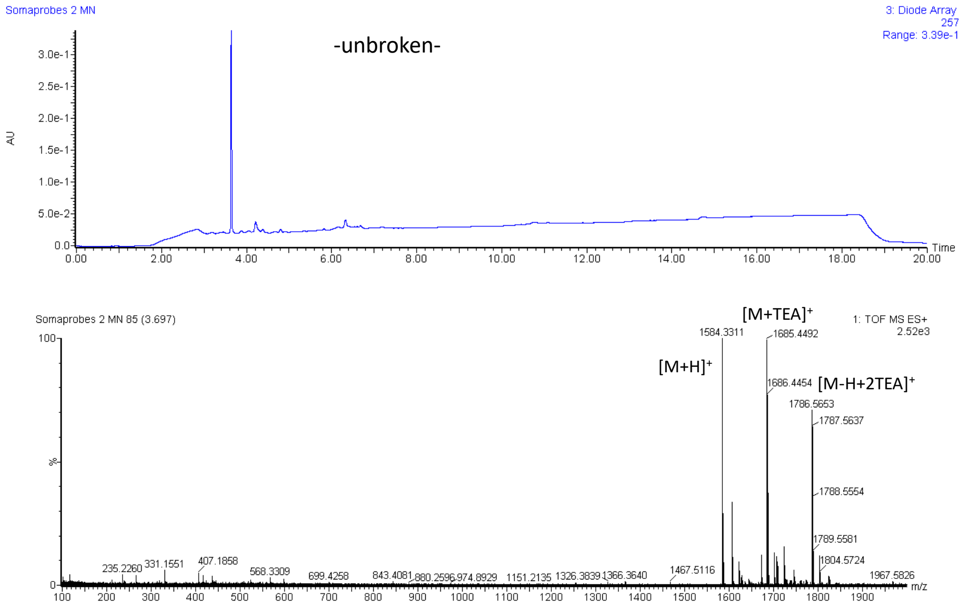


**Figure S14.** UPLC chromatogram (top) and mass spectrum (bottom) of the digestion fragments (none) of pent#2 upon 1h incubation with MNase (λ=257 nm).

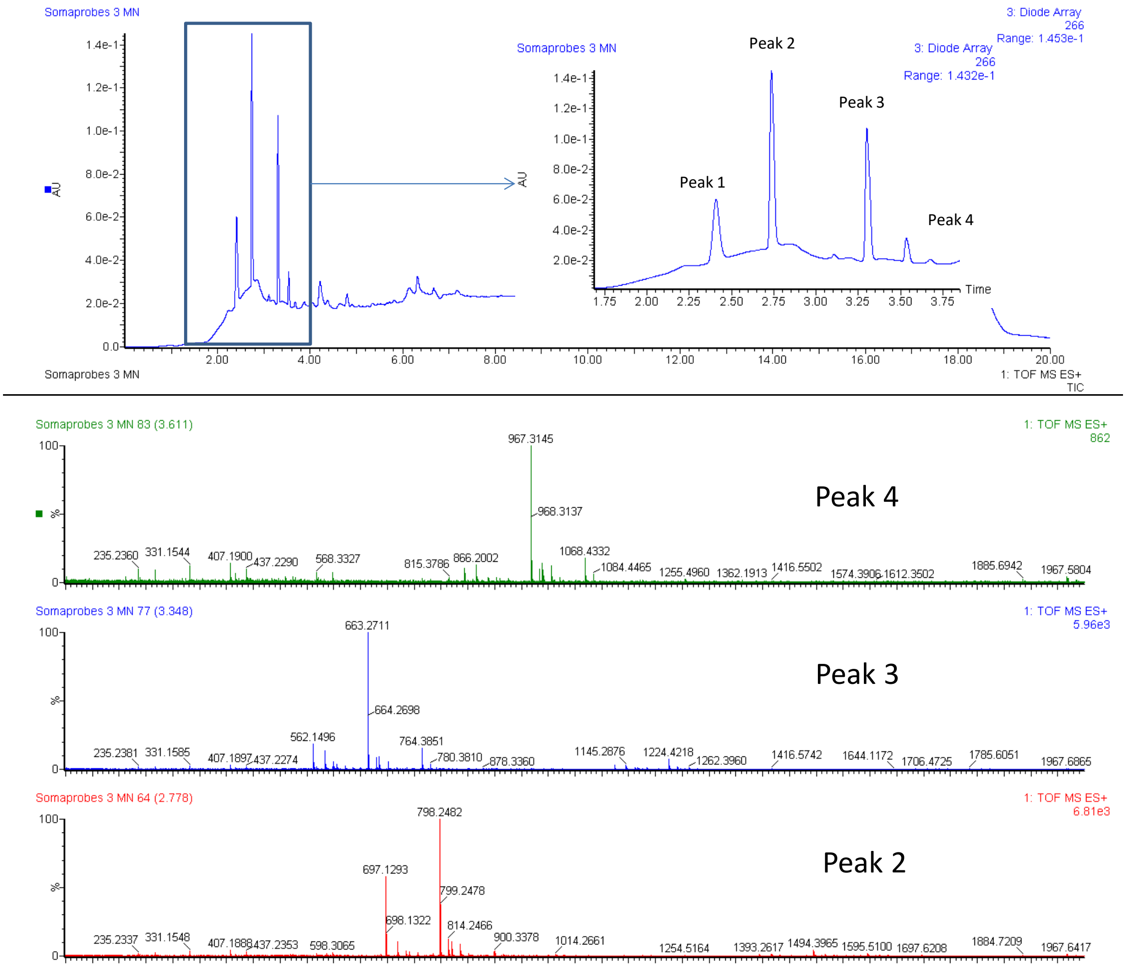


**Figure S15.** UPLC chromatogram (top, λ=266 nm) and mass spectra (bottom) of the digestion fragments of pent#3 upon 1h incubation with MNase.

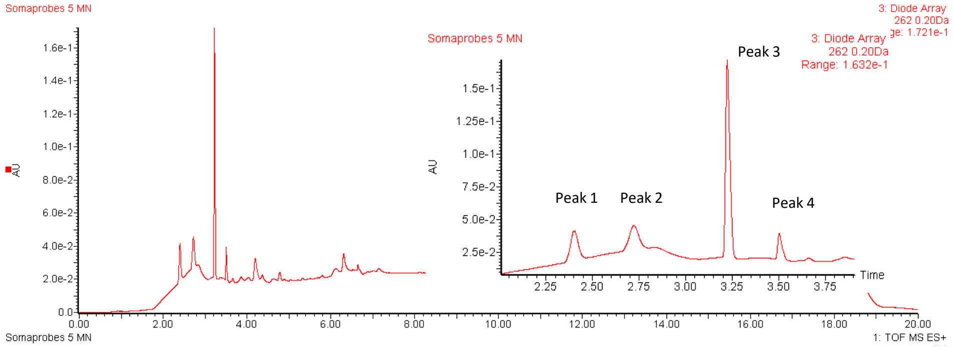


## Sample 4MN ES+

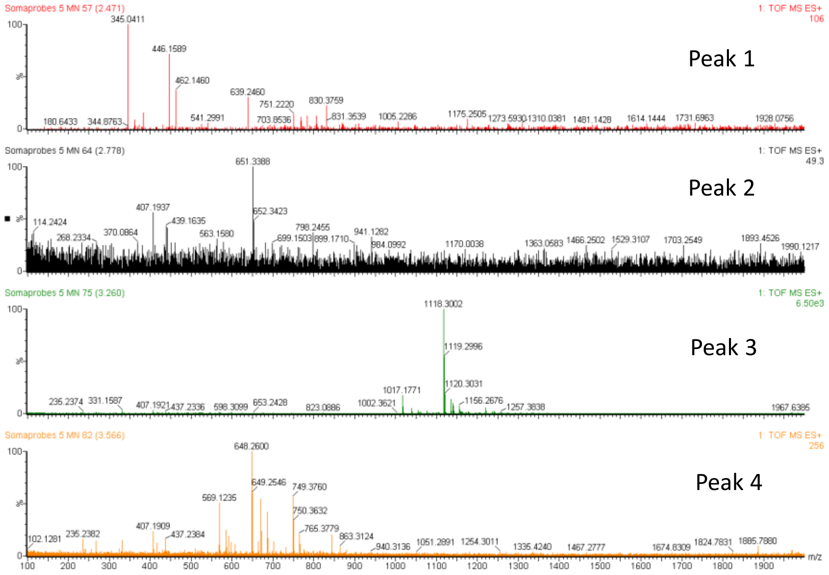


## Sample 4MN ES-

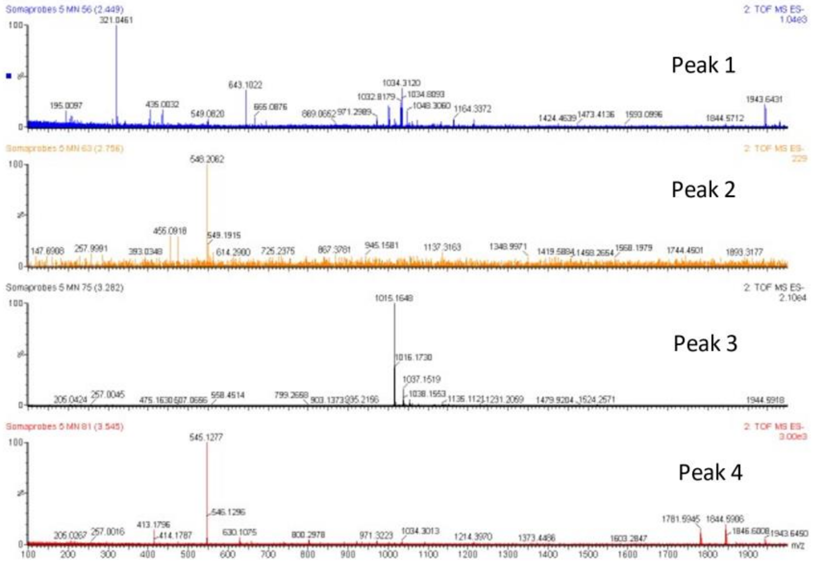


**Figure S16.** UPLC chromatogram (top, λ=262 nm) and mass spectra (middle and bottom) of the digestion fragments of pent#4 upon 1h incubation with MNase.

***Catalytic cycle of the digestion of pentamer 4 (pent#4) upon incubation with MNase.***

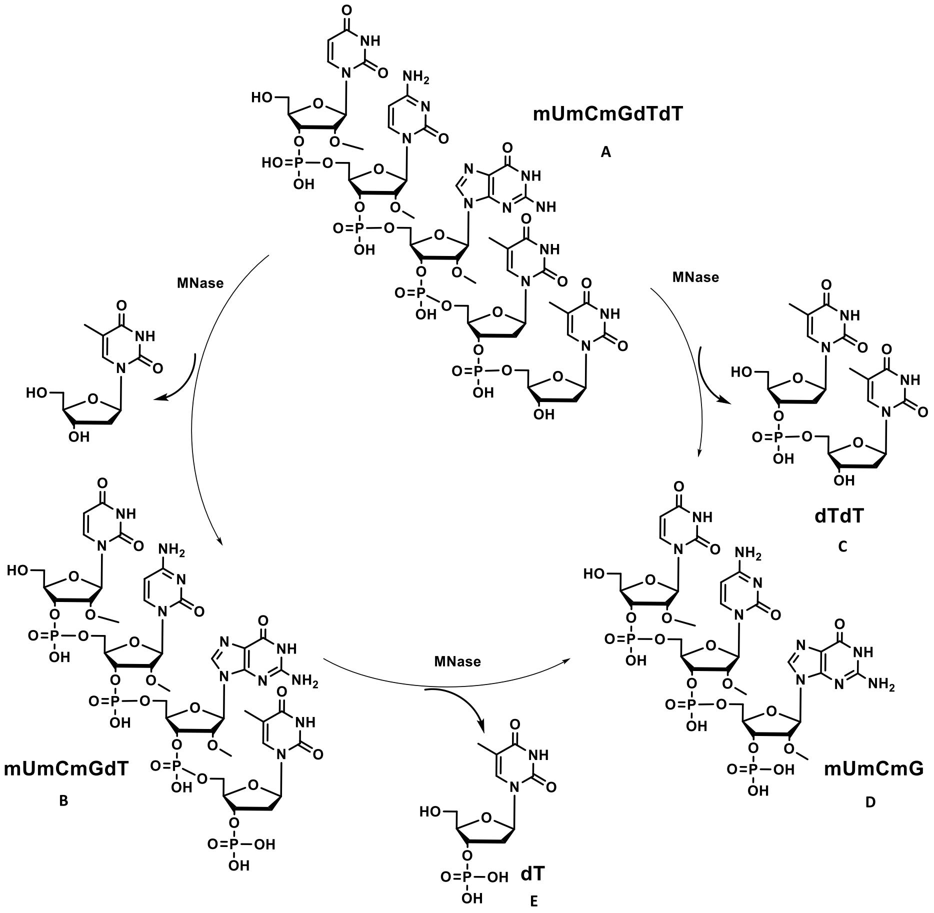


**Figure S17.** Catalytic cycle of the double cleavage process of pentameric oligonucleotide #Pent4 by MNase.

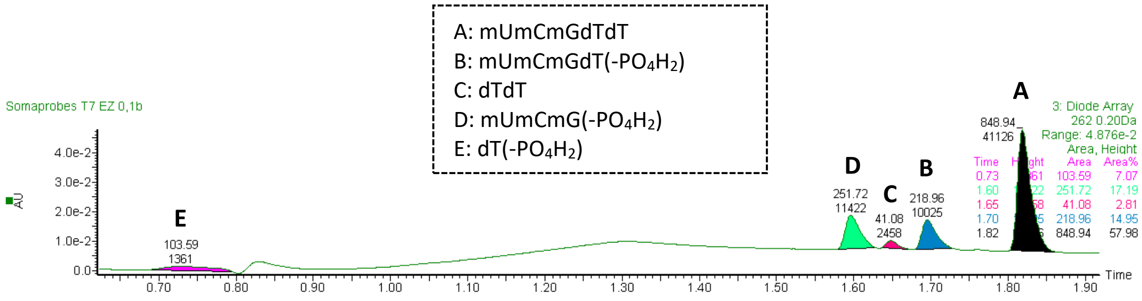


**Figure S18**. Chromatogram of Pent#4 and digestion fragments upon 7 min of incubation with MNase.

***Mass spectra (t = 14 min.).***

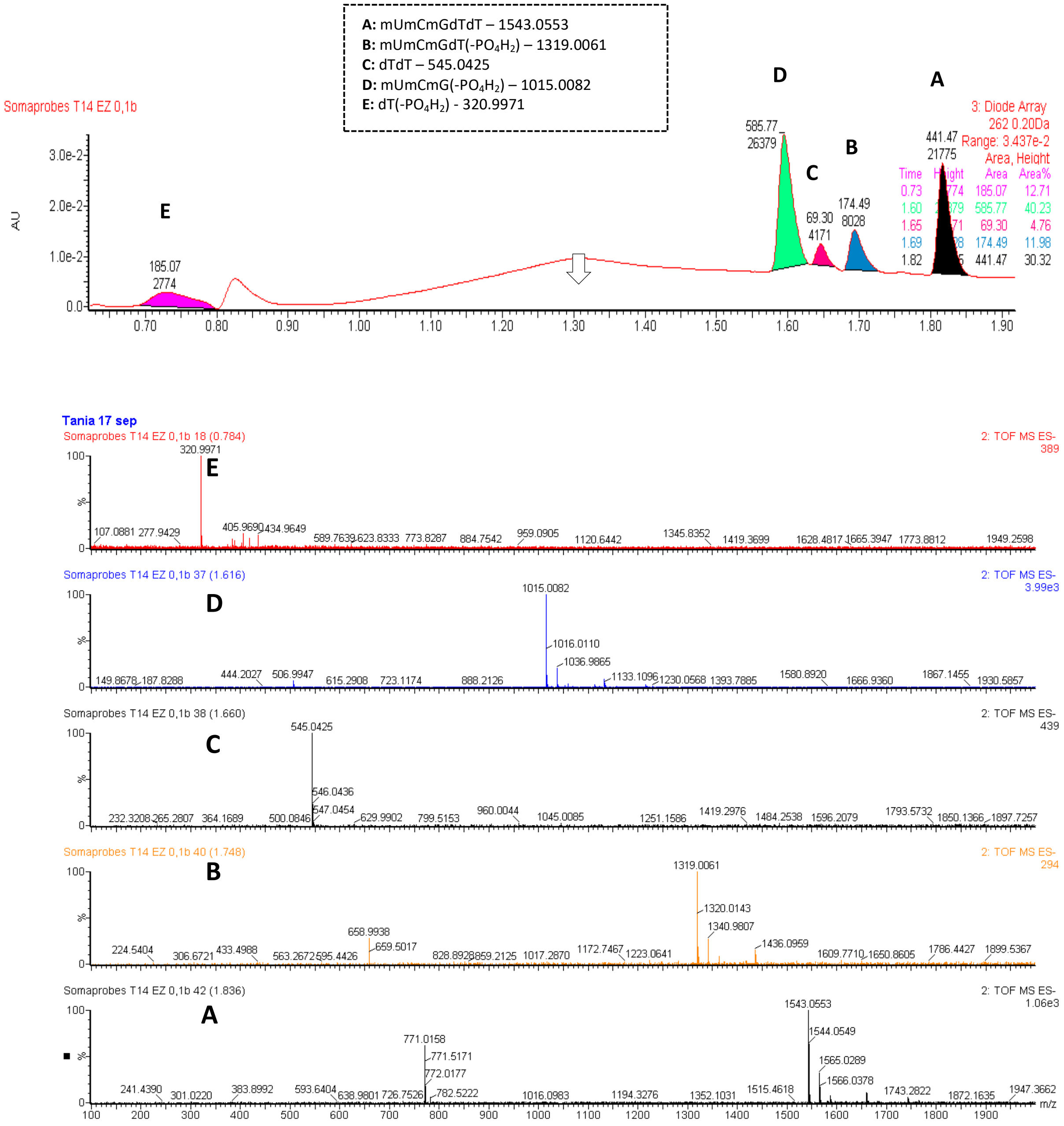


**Figure S19**. Chromatogram (top) of Pent#4 and digestion fragments and (bottom) mass spectra of Pent#4 and fragments at t=14 minutes of incubation.

## ***UPLC-Chromatogramms of pent#4 and digestion fragments at different incubation times (t=0 – t=88 min.)***

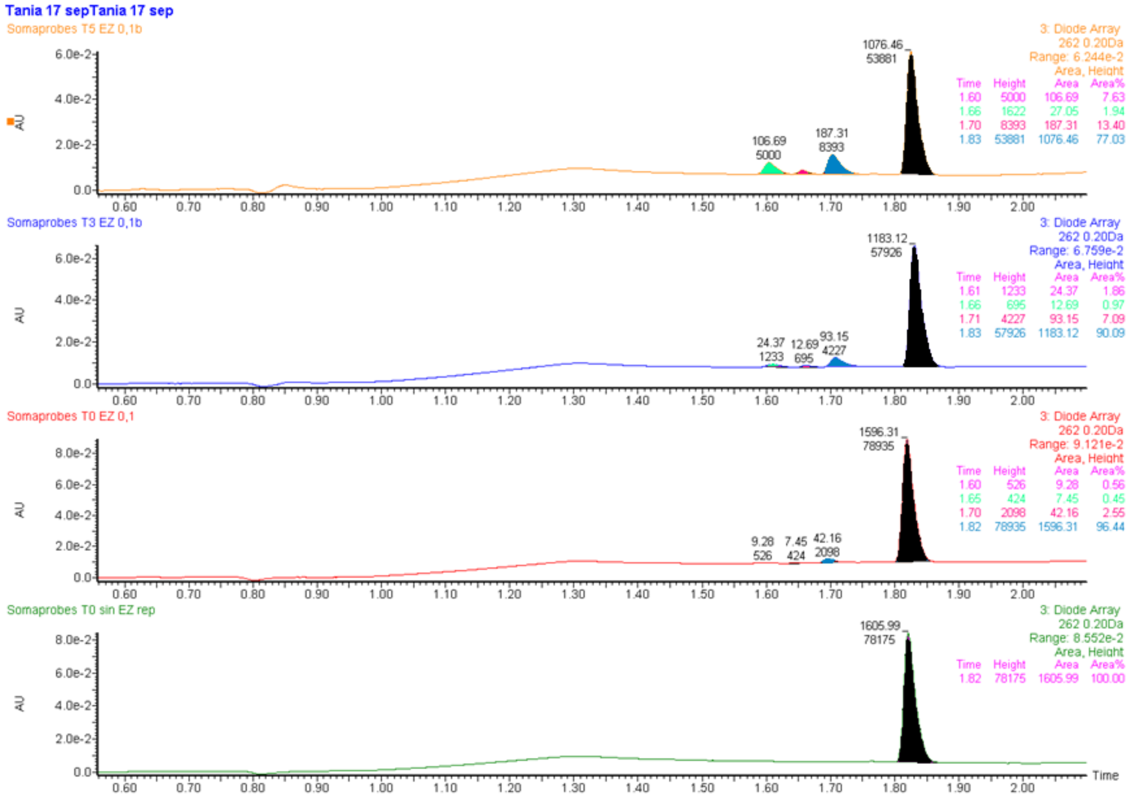


**Figure S20**. UPLC-Chromatograms of Pent#4 and digestion fragments at t=0-5 minutes; λ= 262 nm

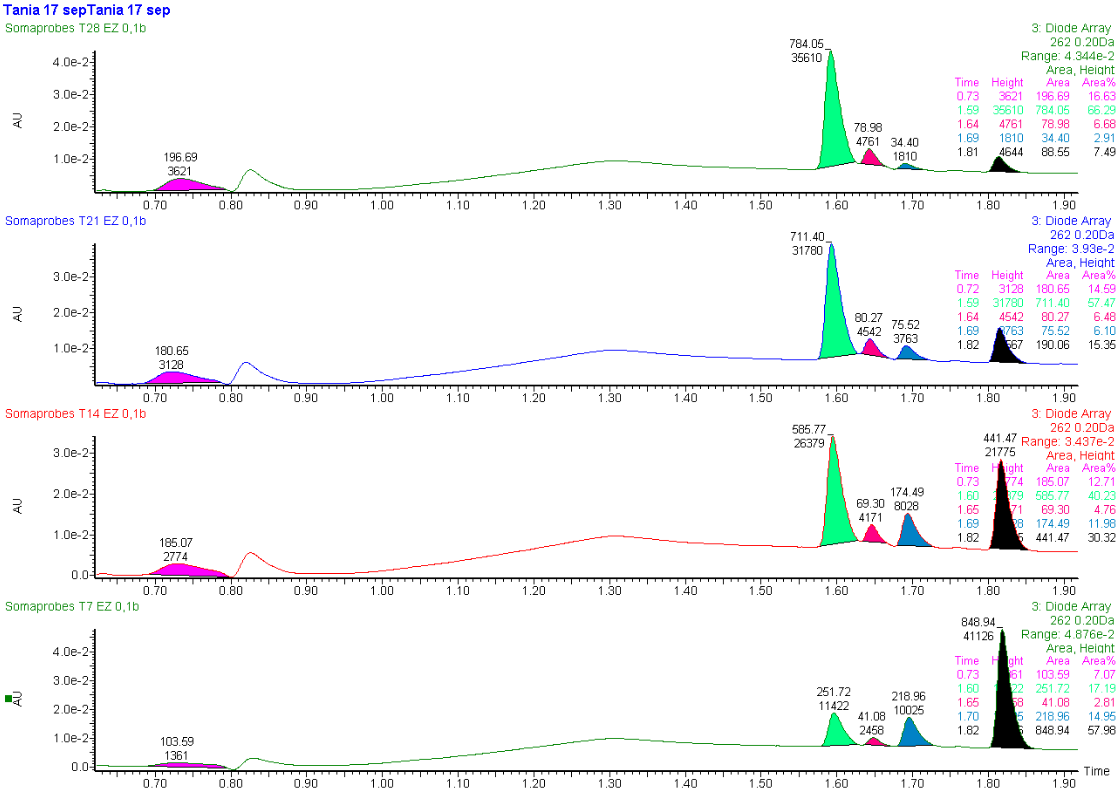


**Figure S21**. UPLC-Chromatograms (top) of Pent#4 and digestion fragments at t=7.26-27.24 minutes; λ= 262 nm

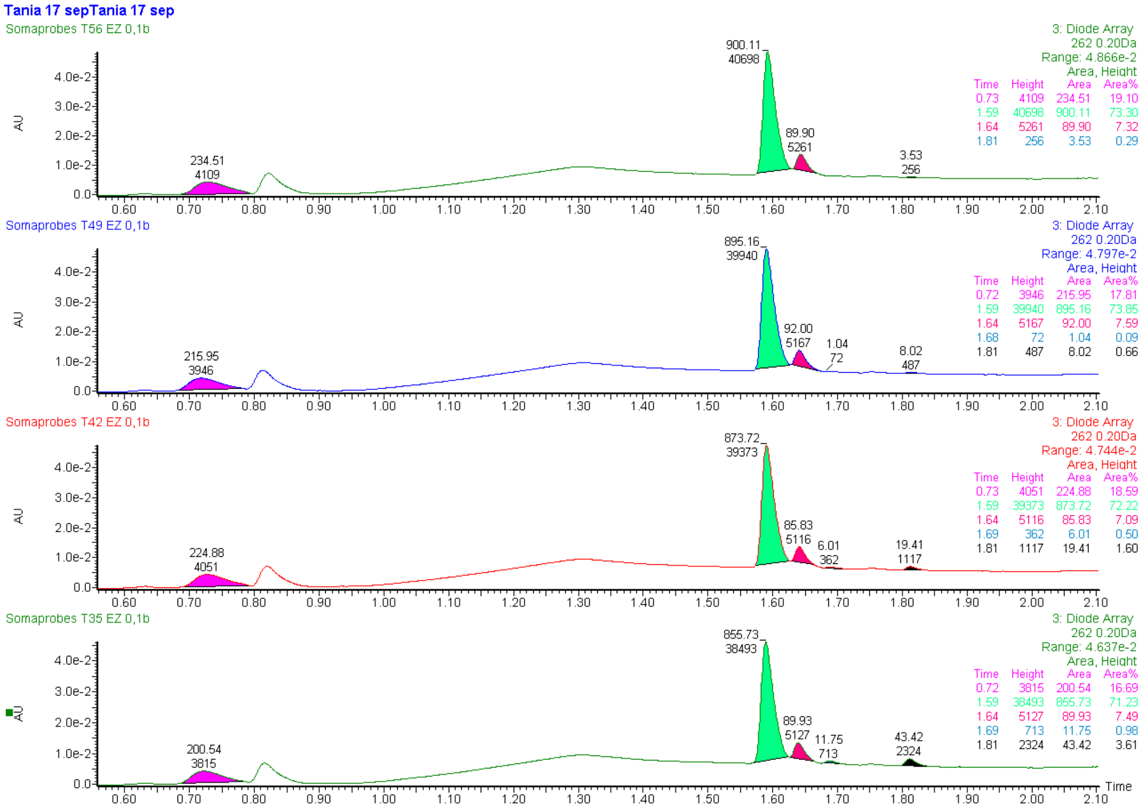


**Figure S22**. UPLC-Chromatograms of Pent#4 and digestion fragments at t=33.9-53.88 minutes; λ= 262 nm

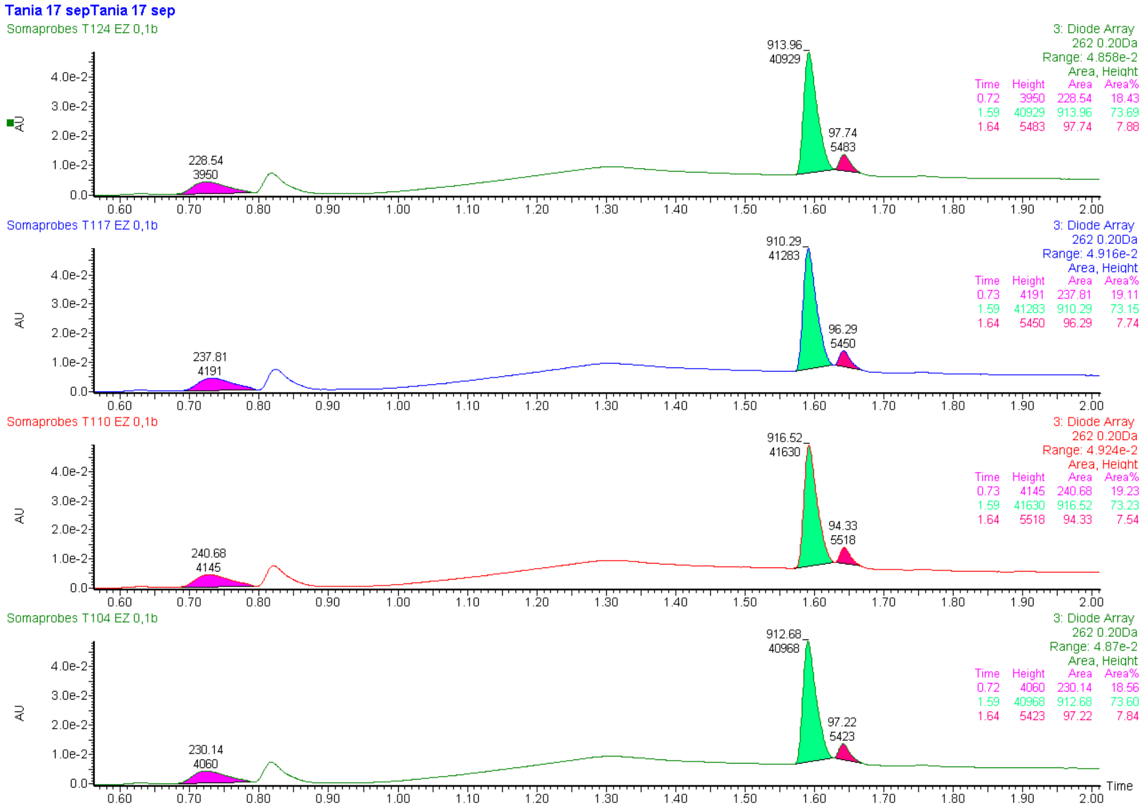


**Figure S23**. UPLC-Chromatograms of Pent#4 and digestion fragments at t=60.54-80.52 minutes; λ= 262 nm

**Kinetics study of the hydrolysis process**

**Table S2.** Kinetics. Pent#4 with MNase (1U/μL) at λ=262nm.

| Fragment | mUmCmGdTdT | mUmCmG | dTdT | mUmCmGdT | dT |
|---|---|---|---|---|---|
| Time (min)/mass(Da) | 1544 | 1016 | 545 | 1320 | 321 |
| 0.00 | 1605 | 0 | 0 | 0 | 0 |
| 0.66 | 1596 | 9 | 7 | 42 | 0 |
| 3.00 | 1183 | 24 | 13 | 93 | 0 |
| 5.00 | 1076 | 107 | 27 | 187 | 0 |
| 7.26 | 849 | 252 | 41 | 219 | 103 |
| 13.92 | 441 | 586 | 69 | 174 | 185 |
| 20.58 | 190 | 711 | 80 | 75 | 181 |
| 27.24 | 88 | 784 | 79 | 34 | 197 |
| 33.90 | 43 | 856 | 90 | 12 | 201 |
| 40.56 | 19 | 874 | 86 | 6 | 225 |
| 47.22 | 8 | 895 | 92 | 1 | 216 |
| 53.88 | 4 | 900 | 90 | 0 | 234 |
| 60.54 | 0 | 913 | 97 | 0 | 230 |
| 67.20 | 0 | 916 | 94 | 0 | 241 |
| 73.86 | 0 | 910 | 96 | 0 | 238 |
| 80.52 | 0 | 914 | 98 | 0 | 228 |

*t=0.00 minutes refers to the experiment without the MNase.
*t=0.00, 3.00 and 5.00 minutes were run independently of the kinetic reaction.

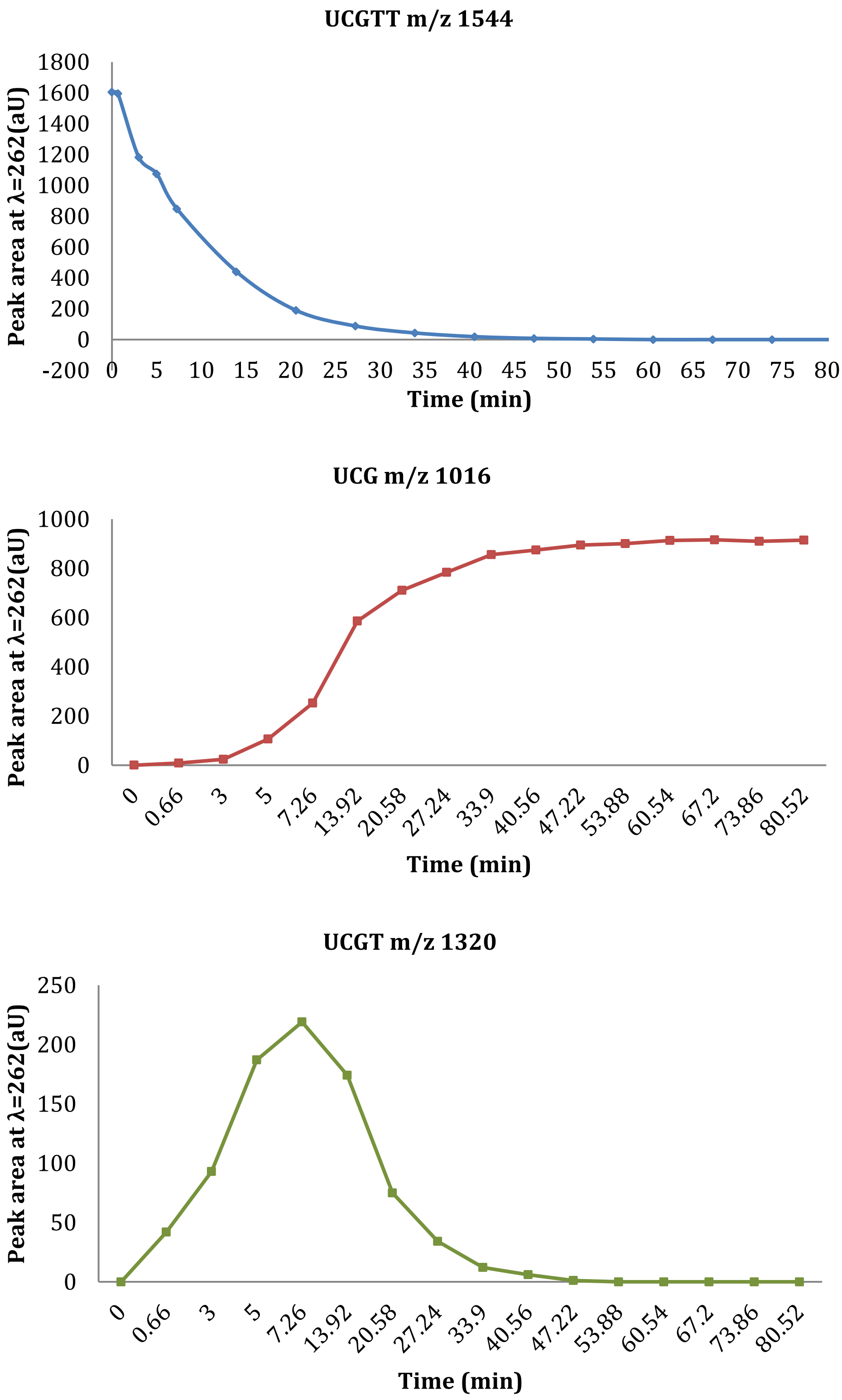


**Figure S24**. Time evolution of the area under the curve of the four peaks identified in the UPLC chromatograms of Pent#4 (mUmCmGdTdT) as well as the digestion fragments generated upon incubation with MNase.

## S7. FRET ASSAYS WITH FLUOROPHORE-LABELLED PENTAMER 4 (FRET-Pent#4)

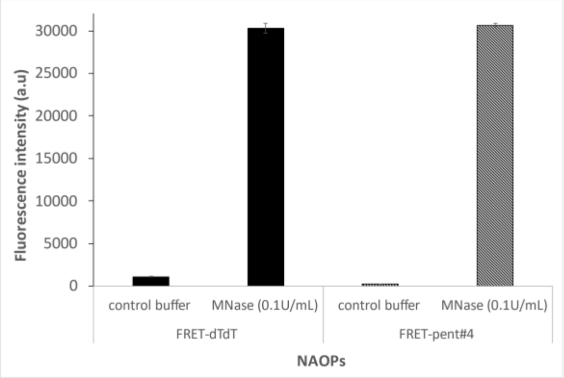


**Figure S25.** Nuclease activity fluorescence profile of FRET-dTdT probe and FRET-pent#4 without and with MNase (0.1U/μL).

Table S3. Raw fluorescence intensity data (±standard deviation).

| *probe* | *Fluorescence intensity (a.u)* | *S. dev. (±)* |
|---|---|---|
| **Control Buffer** | | |
| **FRET-dTdT** | 1102.3 | 41.19 |
| **FRET-Pent#4** | 243.7 | 5.03 |
| **MNase (0.1U/μL)** | | |
| **FRET-dTdT** | 30275.3 | 598.96 |
| **FRET-Pent#4** | 30631.0 | 211.26 |

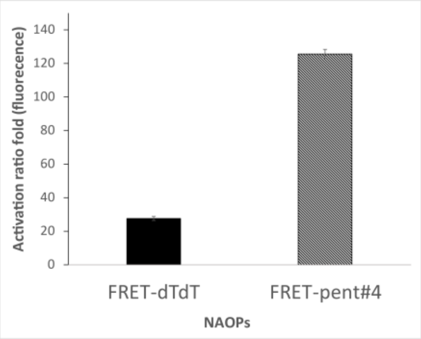


| **Activation ratio fold (ARF)** | |
|---|---|
| **NAOP** | **MNase (0.1U/mL)** |
| **FRET-dTdT** | 27.5 (± 1.16) |
| **FRET-pent#4** | 125.7 (± 2.73) |

**Figure S26.** Activation ratio fold (ARF) of Fluorescence intensity with respect to control for FRET-dTdT probe and FRET-pent#4 obtained from digestion experiments upon incubation with MNase (0.1U/uL).

[1] Loll, P.J., Lattman, E.E. The crystal structure of the ternary complex of staphylococcal nuclease, $Ca^{2+}$, and the inhibitor pdTp, refined at 1.65 A. *Proteins* **1989,** 5**,** 183-201.

[2] Cotton F.A., Hanzen E.E., Legg M.J. Staphylococcal nuclease: Proposed mechanism of action based on structure of enzyme-thymidine 3',5'-bisphosphate-calcium ion complex at 1,5-Å resolution. *Proc. Natl. Acad. Sci. USA*. **1979**, 76(6), 2551-2555.
